# Hierarchical Nanocapsules of Cu-Doped MoS_2_@H-Substituted Graphdiyne for Magnesium Storage

**DOI:** 10.1021/acsnano.1c09405

**Published:** 2022-03-07

**Authors:** Sifei Zhuo, Gang Huang, Rachid Sougrat, Jing Guo, Nini Wei, Le Shi, Renyuan Li, Hanfeng Liang, Yusuf Shi, Qiuyu Zhang, Peng Wang, Husam N. Alshareef

**Affiliations:** † School of Chemistry and Chemical Engineering, Xi’an Key Laboratory of Functional Organic Porous Materials, Northwestern Polytechnical University, Xi’an 710072, PR China; ^‡^Materials Science and Engineering, ^§^Core Labs, and ^∥^Water Desalination and Reuse Center, Biological and Environmental Science and Engineering Division, King Abdullah University of Science and Technology (KAUST), Thuwal 23955-6900, Saudi Arabia; ⊥ Department of Civil and Environmental Engineering, The Hong Kong Polytechnic University, Hong Kong, PR China

**Keywords:** dual-template, hydrogen-substituted graphdiyne, nanocapsule, multiple geometries, rechargeable magnesium battery

## Abstract

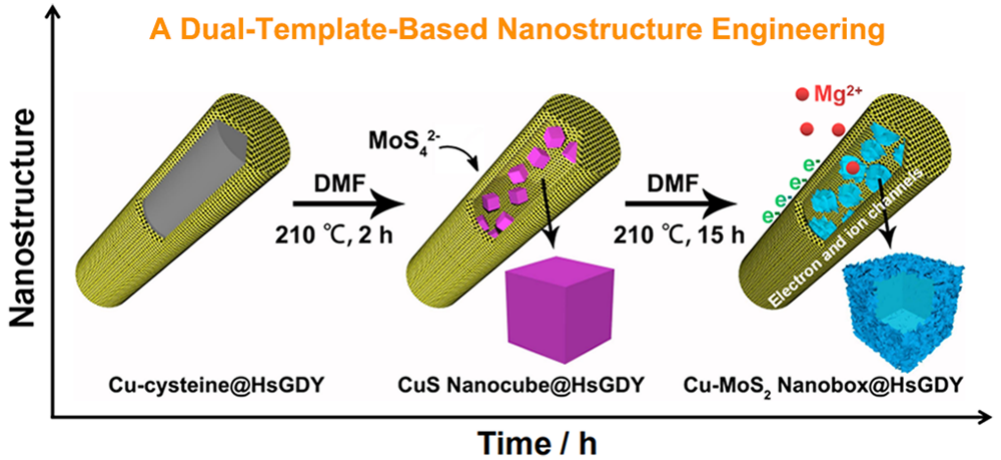

Hierarchical nanocomposites, which integrate electroactive materials into carbonaceous species, are significant in addressing the structural stability and electrical conductivity of electrode materials in post-lithium-ion batteries. Herein, a hierarchical nanocapsule that encapsulates Cu-doped MoS_2_ (Cu-MoS_2_) nanopetals with inner added skeletons in an organic-carbon-rich nanotube of hydrogen-substituted graphdiyne (HsGDY) has been developed for rechargeable magnesium batteries (RMB). Notably, both the incorporation of Cu in MoS_2_ and the generation of the inner added nanoboxes are developed from a dual-template of Cu-cysteine@HsGDY hybrid nanowire; the synthesis involves two morphology/composition evolutions by CuS@HsGDY intermediates both taking place sequentially in one continuous process. These Cu-doped MoS_2_ nanopetals with stress-release skeletons provide abundant active sites for Mg^2+^ storage. The microporous HsGDY enveloped with an extended π-conjugation system offers more effective electron and ion transfer channels. These advantages work together to make this nanocapsule an effective cathode material for RMB with a large reversible capacity and superior rate and cycling performance.

Among the various “beyond Li-ion” battery technologies, rechargeable magnesium batteries (RMB) have attracted strong interest since 2000 in light of the high volumetric capacity (3833 mA h cm^–3^), low reduction potential (−2.37 V vs SHE), and reduced dendrite growth of Mg metal anode in certain electrolyte systems.^1−4^ However, RMB currently lack matching host materials to fill the role of the cathode to improve the sluggish kinetics of Mg^2+^ ions due to the strong electrostatic interactions. For multivalent metal batteries, both recent theoretical and experimental studies have demonstrated that the mobility and intercalation kinetics of multivalent cations are highly dependent on the cathode structure.^5,6^ For this reason, by engineering slit-shaped channels, a 2D layered material with weak interlayer van der Waals interactions provides a powerful platform to construct effective hosts for Mg^2+^ intercalation.^7−10^ Interestingly, as a typical feature of 2D layered MoS_2_, the phase transition from semiconducting 2H-MoS_2_ to metallic 1T-MoS_2_ has been intensively studied to engineer the interlayer channels and activate the basal plane of MoS_2_ at the atomic scale.^11,12^ For example, alkali intercalation (e.g., Li, Na, and K) and heteroatom doping (e.g., Co, Ni, Zn, and O) have been developed for engineering the phase transition of MoS_2_.^13−18^ In contrast to 2H-MoS_2_, the improved electronic conductivity and reduced ion diffusion barrier make 1T-MoS_2_ a strong candidate for RMB, but it has been minimally reported in the literature.^10^ Recently, hydrogen-substituted graphdiyne (HsGDY), a special kind of microporous organic network consisting of benzene rings and butadiyne linkages with an extended π-conjugation system, is arising as a promising support for electrocatalysis, photocatalysis, and organic catalysis.^19−21^ Given its microporous structure with favorable ion diffusion channels, satisfactory electron conductivity, high chemical stability, and easily processable morphology,^22−24^ HsGDY would be a promising “co-host” with MoS_2_ cathode for RMB performance enhancement if we could optimize their integration.

To develop more efficient electrode materials, the nanostructure engineering of multifunctional nanocomposites has been demonstrated both fundamental and technological potential to support the ongoing post-lithium-ion battery technologies. For example, nanocomposites of transition metal-based materials coupled with functional carbonaceous species, such as carbon, graphene, MXene, and graphdiyne, and so on, have been ingeniously developed in some kinds of metal-ion batteries beyond Li-ion batteries.^25−30^ Among them, 2D-layered MoS_2_ yolks skillfully space-confined in hollow carbon shells are emerging as one of the most effective models to optimize their conductivity and accommodate the volume change, but as far as we know, we are never out of trouble with encapsulated MoS_2_ nanosheets being out-of-order.^31,32^ In terms of performance, its attractive properties are offset by the inevitable re-stacking of these disordered nanosheets. To meet this challenge, nanostructural engineering of these disordered MoS_2_ nanosheets into multiple regular geometries would contribute greatly to both their antiaggregation property and enhanced active sites.^33−35^ Despite being attractive in their performance, few effective synthesis technologies exist yet. Although the self-templating strategy has long been known for the direct fabrication of designated nanostructures,^36−38^ no success has been reported in using this approach to derive yolk–shell nanocomposites with inner added geometric skeletons in one continuous process. Thus, developing a conversion mode for self-templating to engineer the MoS_2_ yolks into well-organized nanostructures sealed in the functional HsGDY shell appears particularly intriguing to meet the challenges facing RMB.

Herein, a hierarchical nanocapsule of HsGDY nanotube encapsulated with Cu-MoS_2_ nanopetals and implanted buffer zones (denoted as Cu-MoS_2_@HsGDY) is developed as an effective cathode material for RMB. In the synthesis, a Cu-cysteine hybrid nanowire, which is further conformally coated with a microporous HsGDY layer, is judiciously selected as the precursor. The key point here is that CuS solid nanocubes are first derived from the self-decomposition of Cu-cysteine, and further work uses the subtemplates to derive Cu-MoS_2_ hollow nanoboxes (Figure 1). Specifically, all of these evolutions take place inside of the HsGDY coatings in one continuous process. Such a well-developed Cu-MoS_2_@HsGDY nanocapsule combines the merits of HsGDY (with favorable ion diffusion) and Cu-MoS_2_ (with expanded interlayers and enhanced conductivity). Besides, the well-organized hollow nanoboxes provide numerous inner-added skeletons to accommodate the volume change of Cu-MoS_2_. The rigid HsGDY coating layers with a highly conjugated electronic structure further serve as the electron conductive channel to improve their kinetic activity and structural stability. When evaluated as a cathode material for RMB, it delivers a high reversible charge capacity of 148.5 mAh g^–1^ with excellent cyclic performance (104% capacity retention over 200 cycles) at 50 mA g^–1^. Even at 0.5 A g^–1^, a high capacity of 85.5 mAh g^–1^ is also achieved after 300 cycles. All of these results indicate the strength of organic–inorganic nanocomposites for Mg^2+^ storage.

**Figure 1:**
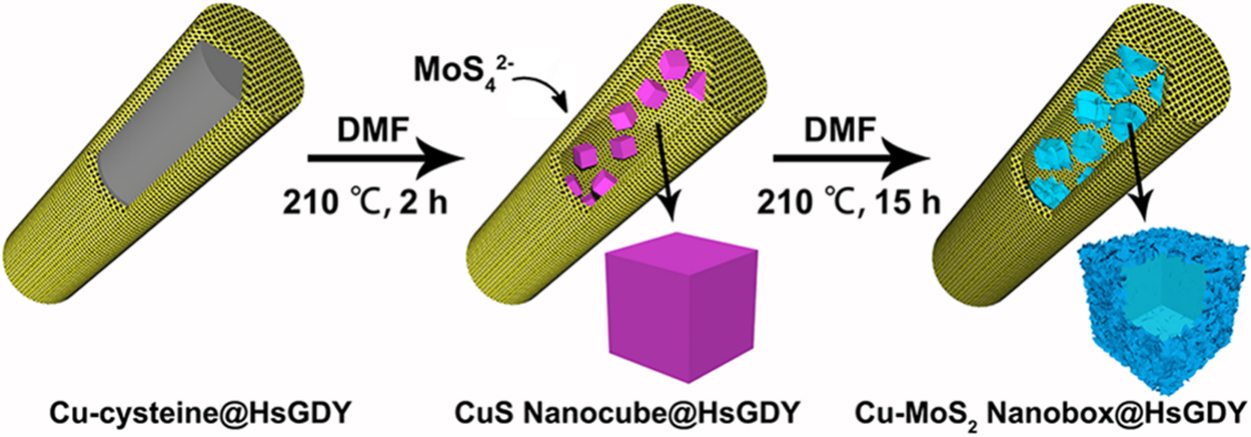
Schematic illustration of the fabrication of hierarchical porous Cu-MoS_2_@HsGDY nanocapsule formed in one continuous process.

## Results and Discussion

### Construction of the Dual Template

**Figure 2:**
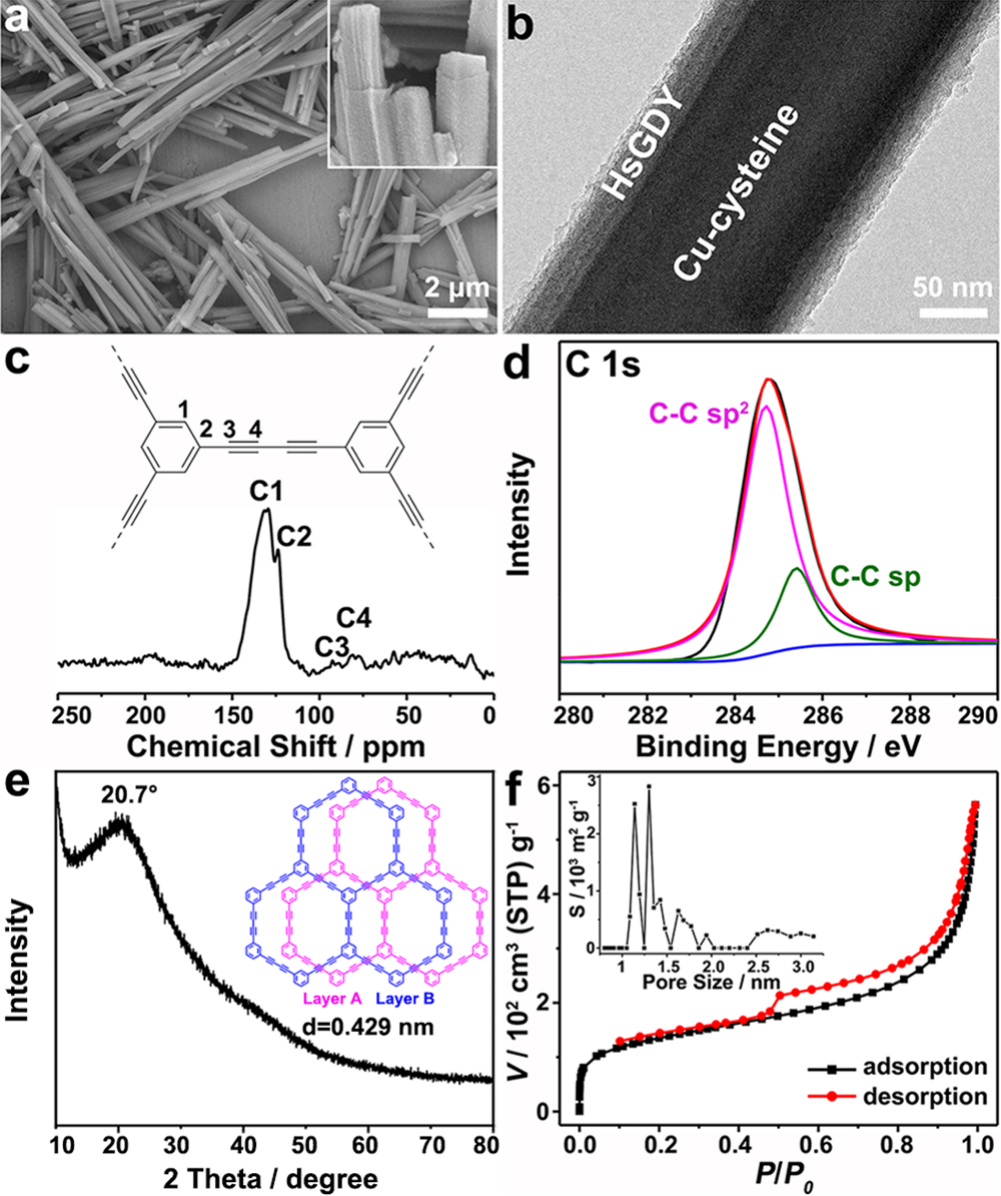
(a) SEM and (b) TEM images of the Cu-cysteine@HsGDY nanowires. (c) Solid ^13^C NMR spectrum, (d) high-resolution XPS spectrum of C 1s, (e) XRD pattern, and (f) N_2_ adsorption/desorption isotherms and the pore size distribution profile analyzed by NLDFT method of the HsGDY nanotubes.

Given that transition metal ions are able to coordinate with biomolecules for exploiting functional nanostructures,^39,40^ we choose Cu^2+^ to coordinate with l-cysteine as the sacrificial template.^41^ As shown in the scanning electron microscopy (SEM) images, highly accessible nanowires with a diameter of around 200 nm and length up to 10 μm are successfully fabricated (Figure S1). Transmission electron microscopy (TEM) characterizations indicate their solid nature with uniform distribution of Cu, C, S, and O (Figure S1). The characteristic vibration peaks, shown in the Fourier transform infrared (FTIR) spectrum and X-ray diffraction (XRD) pattern, further verify the coordination character of Cu^2+^ with l-cysteine, which can be denoted as Cu-cysteine (Figure S2).^41^ In the following step, a conformal coating layer of HsGDY with a thickness of 10 nm is seamlessly cross-linked on the surface of these nanowires by a Glaser coupling reaction of 1,3,5- triethynylbenzene (Figures 2a and S3).^21^ The clear and continuous boundaries between Cu-cysteine and HsGDY verify the conformal coating nature of HsGDY without any influence on the Cu-cysteine, which is further confirmed by the consistent results of FTIR, XRD and scanning TEM-electron energy loss spectroscopy (STEM-EELS) before and after HsGDY coating (Figurse 2b, S2, and S4). Herein, the reactivity of l-cysteine makes it an *in situ* sulfur source confined in the HsGDY capsule.

To collect the chemical structure of HsGDY, Cu-cysteine cores are selectively etched by acid (Figure S5). Consequently, the solid ^13^C nuclear magnetic resonance (NMR, Figure 2c) spectrum indicates the large π-conjugation system of HsGDY bearing sp-hybridized alkyne (δ = 90.3 and 81.5 ppm) and sp^2^-hybridized aryl (δ = 136.2 and 123.1 ppm).^21,24^ The C 1s spectrum of HsGDY analyzed by the X-ray photoelectron spectroscopy (XPS, Figure 2d) can be also deconvoluted into two typical fitting curves of C=C (sp^2^) at 284.7 eV and C≡C (sp) at 285.4 eV.^21,24^ Besides, the characteristic vibration peaks of 1370 and 1597 cm^–1^ shown in the Raman spectrum are assigned to the D and G bands of sp^2^ carbon domains in the HsGDY capsules (Figure S6a).^21,24^ The XRD pattern further affirms the layered structure of HsGDY with an interlayer spacing of 4.29 Å (Figures 2e and S6). Altogether, HsGDY is a carbon-rich framework comprised of benzene rings connected with butadiyne linkages with a formula of nC_72_H_18_.^24^ Its extended π-conjugated structure qualifies as a conductive additive for electrode materials to satisfy their conductivity. Additionally, a microporous structure with a pore size of around 1.2 nm and a specific surface area of around 465 m^2^ g^–1^ is also generated during the cross-coupling process of 1,3,5-triethynylbenzene (Figure 2f). The difference between the theoretical (1.6 nm) and experimental values (1.2 nm) of the micropores suggests the AB stack mode of HsGDY layer (Figure S7). Such HsGDY networks could serve as physical capsules with numerous ion channels to confine the *in situ* chemical conversion of Cu-cysteine.

### Synthesis of the Hierarchical Porous Nanocapsule

**Figure 3:**
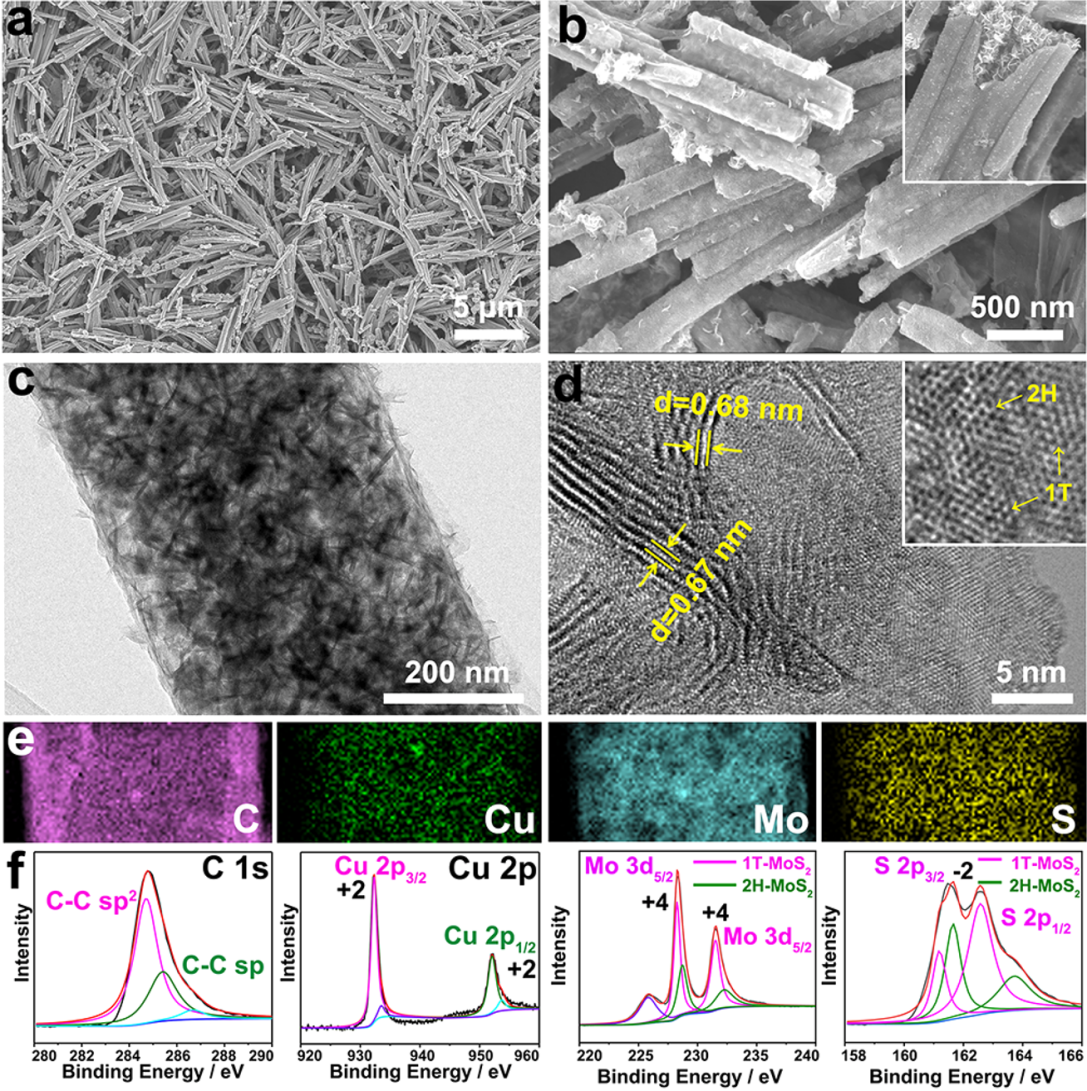
(a, b) SEM images, (c) TEM image, (d) HRTEM images, (e) Side-view STEM-EELS elemental mapping, and (f) High-resolution XPS spectra of the Cu-MoS_2_@HsGDY nanocapsule.

Hence, these Cu-cysteine@HsGDY nanowires are subjected to reaction with (NH_4_)_2_MoS_4_. As shown in the SEM and TEM images (Figures 3a–c and S8), some gorgeous nanopetals instead of Cu-cysteine nanowires are solely confined in a capsule. Their evident lattice fringes with a distance around 0.68 nm suggest the dominated formation of hexagonal MoS_2_ with expanded interlayers (Figure 3d).^15,32^ Besides, the metallic 1T-MoS_2_ structure coexists with the 2H-MoS_2_ phase in Cu-MoS_2_@HsGDY, resulting from the Cu heteroatom doping (Figure 3d). In line with the HRTEM result, the high-resolution XPS spectrum of Mo 3d can be deconvoluted into four primary peaks at 228.3 eV (Mo 3d_5/2_) and 231.5 eV (Mo 3d_3/2_) for 1T-MoS_2_ and 228.7 eV (Mo 3d_5/2_) and 232.3 eV (Mo 3d_3/2_) for 2H-MoS_2_, respectively, which further evidence the coexistence of 1T and 2H phases in Cu-MoS_2_@HsGDY (Figures 3f and S9).^15,42^ Besides MoS_2_, STEM-EELS elemental mappings clearly reveal their homogeneous incorporation with elemental Cu (Figure 3e). The valence state of Cu^2+^ is further confirmed by the presence of binding energies of Cu 2p_3/2_ (932.3 eV) and Cu 2p_1/2_ (952.1 eV) shown in the Cu 2p orbital (Figure 3f). Inductively coupled plasma-atomic emission spectrometry (ICP-AES) suggests the atomic ratio of Cu/Mo is around 0.83, which agrees well with the energy dispersive spectroscopy (EDS) result (Cu/Mo = 0.85, Figure S10). As no CuS phase is detected in both HRTEM image and XRD pattern (Figures 3d and 5i), these nanopetals can be identified as Cu-MoS_2_.^43^ Although a thorough transformation has happened on the Cu-cysteine, both chemical structure and morphology of the HsGDY coating are left intact as evidenced by the consistent of the C 1s XPS, solid ^13^C NMR and FTIR spectra (Figure 3f, S11, and S12), as well as the tubular distribution of C shown in the STEM-EELS elemental mapping (Figure 3e). By calculation based on the thermogravimetric analysis (TGA), the weight percentage of HsGDY in Cu-MoS_2_@HsGDY is estimated to be 10 wt % (Figure S13). As a whole, the specific surface area and pore volume of these Cu-MoS_2_@HsGDY capsules are calculated to be 168 m^2^ g^–1^ and 0.627 cm^3^ g^–1^, respectively (Figure S14). Notably, besides micropores of HsGDY, two typical mesopores (12 and 34 nm) also exist (Figure S14), which provide considerable diffusion channels and contact area for the electrolyte.^44^ All of these results verify that the Cu-cysteine nanowires are *in situ* transformed into Cu-MoS_2_ nanopetals in the confined HsGDY capsule, which remains fixed.

**Figure 4:**
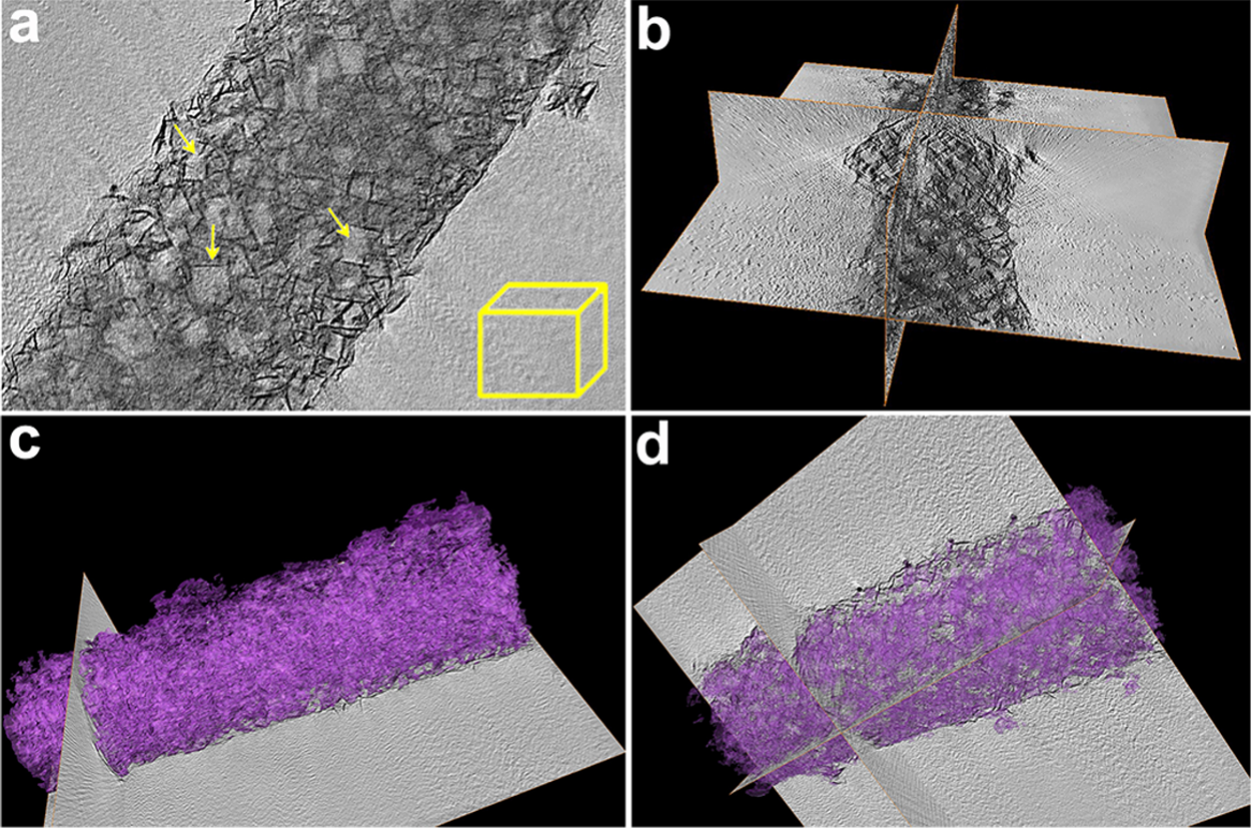
TEM tomography study: virtual cross section (a), 3D reconstruction (b), 3D volume (c, d) of the hierarchical porous Cu-MoS_2_@HsGDY nanocapsule.

To collect better structural information on the internal Cu-MoS_2_ nanopetals, 3D electron tomography is adopted to visualize their arrangement, which cannot be observed under 2D microscopy (Video S1).^45^ Interestingly, many squares are shown in the typical virtual cross section taken from the original 3D tomograms (Figures 4a and S15). When constructing these series of virtual cross sections along the *Z*-axis through the sample captured from −65 to 65° at 2° initial intervals, a video of 3D tomography is acquired (Video S2). Accordingly, it is found that many regular hollow nanoboxes with side lengths around 30–50 nm are embedded in these gorgeous nanopetals, which is consistent with the pore-size distribution shown in the BET result (Figure S14). A three-plane view of *XY*, *XZ*, and *YZ* reconstructed from these cross sections also illustrates that many cubic cavities are surrounded by numerous nanopetals (Figure 4b). These hollow nanoboxes provide internal-added skeleton geometries to prevent the nanopetals from aggregation, which is crucial for performance enhancement. Besides, the derived segmented volumes clearly demonstrate the blooming mesoporous structure with internal-connected channels, which not only offer more exposed active sites but also facilitate mass transfer (Figures 4c,d and S15). Therefore, these hierarchical nanocapsules of Cu-MoS_2_@HsGDY successfully incorporate functional organic species with electroactive inorganic material with multiple geometries. All of these features would work well together in synergy to make this integrated system a promising candidate for RMB with high structural stability and mass transfer capability.

**Figure 5:**
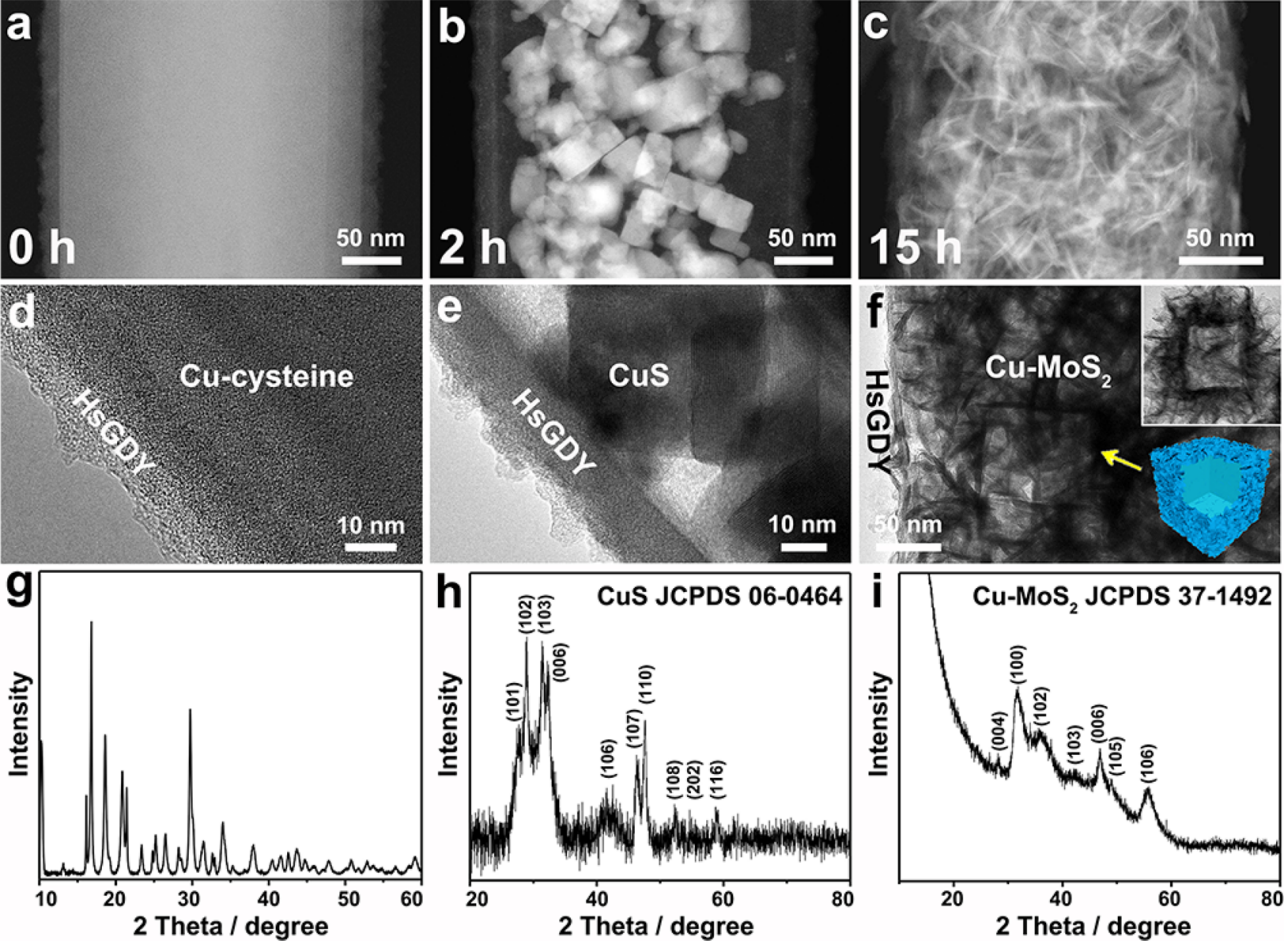
(a–c) STEM images, (d–f) TEM images, and (g–i) XRD patterns of the intermediates collected at different reaction stages in the continuous process: 0 h (a, d, g), 2 h (b, e, h), 15 h (c, f, i). PDF nos. 06–0464 and 37–1492, Joint Committee on Powder Diffraction Standards.

For understanding the morphology/crystal evolution clearly in the continuous process, several intermediates are collected at different reaction stages. Interestingly, some regular nanocubes with a size of around 30–50 nm solely confined in the HsGDY are identified in the first 2 h (Figure 5b,e and Figure S16). The XRD pattern (Figure 5h, Powder Diffraction File (PDF) no. 06–0464, Joint Committee on Powder Diffraction Standards (JCPDS)) and STEM-EELS elemental mappings (Figure S16) indicate these nanocubes as cubic CuS.^46,47^ To clarify this crystal transition, Cu-cysteine and Cu-cysteine@HsGDY are separately subjected to the same solvothermal treatment but without (NH_4_)_2_MoS_4_. As expected, some discrete CuS nanocubes and CuS nanocubes confined in HsGDY (CuS@HsGDY) are generated, respectively (Figure S17). Besides, Cu-MoS_2_ nanoboxes are also generated when we use Cu-cysteine to react with (NH_4_)_2_MoS_4_ (Figure S18). These results directly verify that these CuS nanocubes are derived from the self-decomposition of the Cu-cysteine. Accordingly, the selection of l-cysteine is judicious, as it first coordinates with Cu^2+^ to form an initial 1D template (Figure 5a,d,g), and then serves as the *in situ* sulfur source to evolve CuS intermediates (Figure 5b,e,h). Then, these CuS nanocubes act as the secondary self-template to derive the ultimate nanosheet-based Cu-MoS_2_ nanoboxes by reaction with the *ex situ* (NH_4_)_2_MoS_4_ (Figure 5c,f,i). It has been reported that the presence of transition metal ions during the nucleation of MoS_2_ could bond to the free sulfur, which disrupts the regular atomic arrangement in MoS_2_ by formation substitutional defects.^15^ Therefore, we proposed that the Cu^2+^ dissolved from the CuS nanocubes may suppress the growth of MoS_2_ crystal along the basal planes and simultaneously incorporate into MoS_2_ to form Cu–Mo–S phase by substituting on Mo sites. The mismatch in bond length between Cu and Mo atoms initiates the phase transition of MoS_2_ near the defect sites, which accounts for the coexistence of 2H-MoS_2_ and 1T-MoS_2_.^15^ Besides nanoboxes shown in the nanocapsules, some discrete nanoboxes are also detected from the broken areas, which directly illustrate the internal geometries of Cu-MoS_2_ as well (Figure 5f). Notably, all of these evolutions in both morphology and crystal changes happen in one continuous process. Since the inner added nanoboxes take shape *in situ* without any support from extra added templates, this method is indeed simple and cost-effective. In addition, by selecting suitable metal ions to coordinate with some special biomolecules or ligands, it is possible to access various electrode materials with improved composition/structure-dependent performance, for instance, M-MoS_2_@HsGDY (M = Fe, Co, Ni, etc.)^21^ and MS_*x*_@HsGDY (M = Fe, Co, Ni, Ti, V, Sn, etc.).^48^

### Electrochemical Performance for RMB

**Figure 6:**
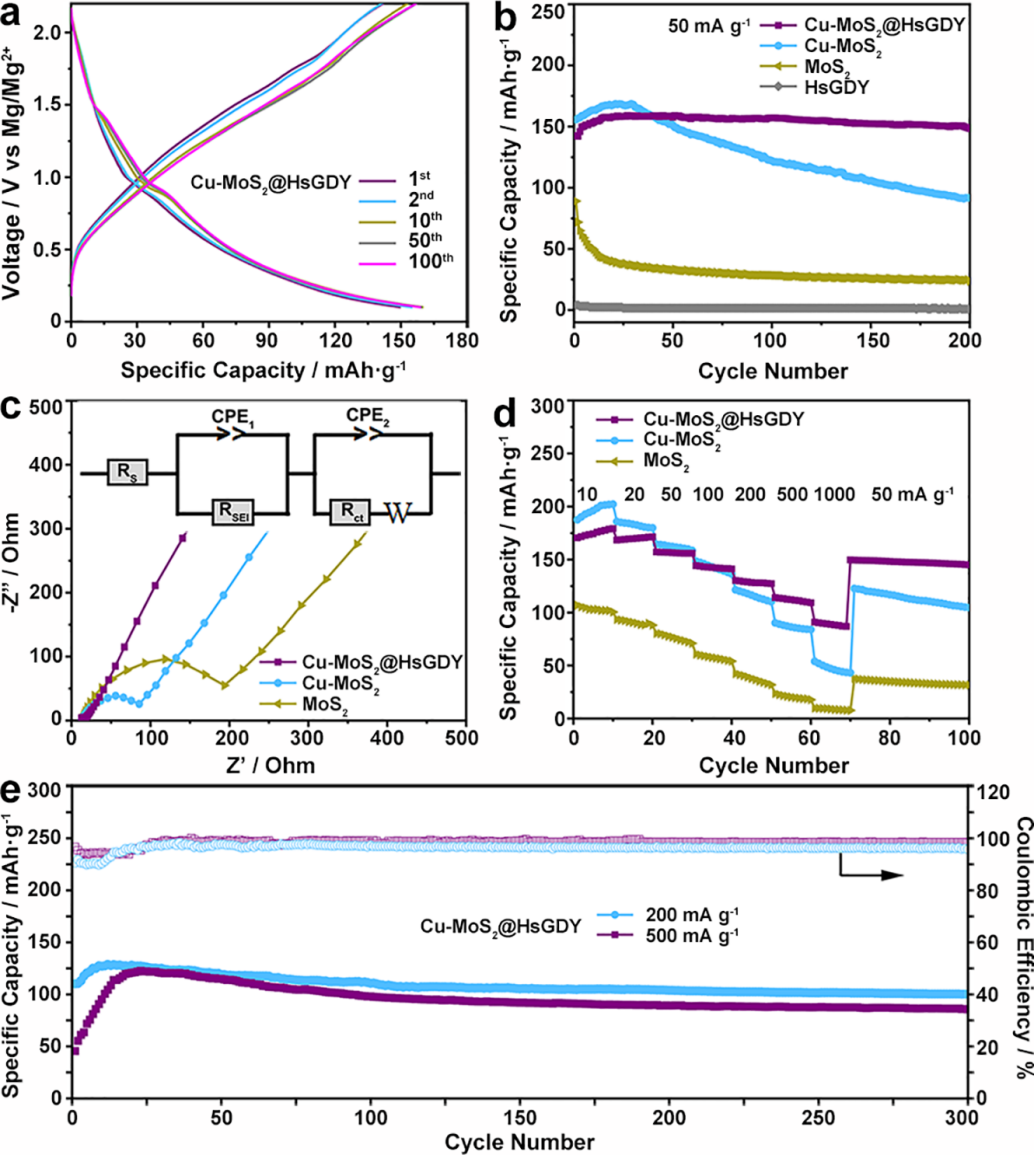
(a) Discharge/charge profiles of Cu-MoS_2_@HsGDY at 50 mA g^–1^. (b–d) Cycling performance (b), Nyquist plots (c), and rate capability (d) of Cu-MoS_2_@HsGDY, Cu-MoS_2_, MoS_2_, and HsGDY. (e) Cycling performance of Cu-MoS_2_@HsGDY at 200 and 500 mA g^–1^.

Given their sufficient electron/ion conductivity and multiple geometric skeletons, these Cu-MoS_2_@HsGDY nanocapsules are evaluated as cathode materials for RMB. For comparison, HsGDY nanotubes, MoS_2_ nanospheres, and Cu-MoS_2_ nanoboxes are also tested (Figures S5, S18, and S19). The cyclic voltammetry (CV) curves of Cu-MoS_2_@HsGDY (Figure S20) with main redox peaks at around 0.9/1.8 V vs Mg/Mg^2+^ suggest the reversible intercalation/deintercalation of Mg^2+^ into Cu-MoS_2_ interlayers.^10^ Consistent with the CV results, two small plateaus at 0.9 and 1.8 V vs Mg/Mg^2+^ are also shown in the typical galvanostatic discharge–charge profiles, respectively (Figures 6a and S21). The diffraction peaks of Cu-MoS_2_@HsGDY are well-preserved, and no other species are generated along with the discharge–charge process. It is worth noting that the peaks shift toward low and high angles, respectively, in the discharge and charge states, which suggests the intercalation mechanism of Cu-MoS_2_ without conversion reaction (Figure S22).^49^ Besides, the sharply increased Mg content is shown in the *ex situ* XPS and TEM-EDS mapping after discharging, which is then further significantly decreased in the subsequent charge process, clearly suggests the Mg^2+^ ions storage in the Cu-MoS_2_ host (Figures S23 and S24). Thereinto, no metallic Mo appears in the discharge–charge process, which is further evidence that no conversion reaction happens. However, it is proposed that a phase transition between 2H-MoS_2_ and 1T-MoS_2_ happens upon cycling (Figure S23). It should be noted that no valence change takes place in the doped Cu^2+^ (Figure S23), and it is believed that the metallic Mo–S phase after Cu^2+^ doping facilitates the electron transfer.^14−18^ As a result, the Cu-MoS_2_@HsGDY nanocapsules deliver a high initial discharge capacity of 150 mAh g^–1^ with a Coulombic efficiency (CE) of 95%, which is larger than most of the reported MoS_2_-based materials (Table S1). In the initial cycles, the Cu-MoS_2_@HsGDY nanocapsules delivers a lower discharge capacity (142 mAh g^–1^) than Cu-MoS_2_ nanoboxes (156 mAh g^–1^), 10% to be exact. However, it should be noted that the weight percentage of HsGDY in Cu-MoS_2_@HsGDY is estimated to be 10 wt %, which suggests that the active component of Cu-MoS_2_ contributes equally in stoichiometry. Having said that, the presence of HsGDY replaces some of the active mass, but is worth it in terms of kinetics. As a result, a reversible charge capacity of 148.5 mAh g^–1^ with a high capacity retention of 104% is achieved in cycle 200 (Figure 6b), which compares favorably against Cu-MoS_2_ nanoboxes (91.7 mAh g^–1^, 59%) and MoS_2_ nanospheres (23.5 mAh g^–1^, 26.4%). For one reason, the fluffy nanopetals of Cu-MoS_2_ with expanded interlayers provide abundant exposed active sites to host Mg^2+^ ions with improved diffusion kinetics. For another, the multiple skeleton geometries comprising of outer rigid HsGDY capsules and inner-added rectangular nanoboxes work together to reduce restacking and mitigate aggregation of the confined Cu-MoS_2_ nanopetals (Figures S25 and S26). All of these merits contribute greatly to the specific capacity of Cu-MoS_2_@HsGDY.

Although they lack Mg^2+^ storage capability, the conjugated microporous HsGDY capsules can further work as an effective electron/ion channel to improve the kinetics of the cathode. As shown in the fitted Nyquist plots (Figures 6c and S27), all of the MoS_2_, Cu-MoS_2_, and Cu-MoS_2_@HsGDY cathode materials exhibit similar features with one depressed semicircle in the high-medium-frequency region (refer to charge-transfer resistance *R*_ct_ and SEI film resistance *R*_SEI_) and an oblique line in low-frequency region (refer to Warburg impedance W related to the Mg^2+^ diffusion), respectively. As revealed by the equivalent circuit diagrams (Figure 6c and Figure S27), the values of *R*_ct_ decrease significantly from 111.70 Ω to 57.76 Ω when MoS_2_ was doped with Cu heteroatoms (Cu-MoS_2_), suggesting much-facilitated electron transfer by introducing transition metal heteroatoms in MoS_2_. A further decreased *R*_ct_ value of Cu-MoS_2_@HsGDY (9.83 Ω) was obtained by the incorporation of the HsGDY nanocapsules, which indicates the advantage of HsGDY as effective electron-conductive channels during the charge/discharge processes. In addition, when compared with Cu-MoS_2_ (0.03931 Ω s^–1/2^), the lower *W* value of Cu-MoS_2_@HsGDY (0.01363 Ω s^–1/2^) further revealed the function of HsGDY as ion channels to facilitate electrolyte penetration. To reveal the diffusion kinetics of Mg^2+^ ions in electrode material, the Mg^2+^ diffusion coefficient (*D*_Mg^2*+*^_) is utilized to quantify their comparative kinetic effectiveness (Figure S27d). As a result, after doping with Cu heteroatoms, the Mg^2+^ diffusion coefficient for Cu-MoS_2_ is 1.76 times higher than that of MoS_2_, which directly suggests the function of Cu–Mo–S phase which facilitated Mg^2+^ diffusion. For one thing, the enlarged slit-shaped channels along the Cu-MoS_2_ layers from edge to bulk provides much more efficient diffusion access for Mg^2+^ ions.^50^ For another, the presence of 1T-MoS_2_ phase with higher intrinsic conductivity contributes to the faster transfer of Mg^2+^. When Cu-MoS_2_ is further encapsulated in the HsGDY coating, the Mg^2+^ diffusion coefficient is nearly doubled in the mode of Cu-MoS_2_@HsGDY over that of Cu-MoS_2_. In such a case, the HsGDY capsule could serve as the ion-buffer reservoirs to keep a steady flow of electrolyte, while the built-in skeletons facilitate ion diffusion across the whole bulks, both of which contribute to the improved Mg^2+^ diffusion coefficient.^51^ Besides, the fluffy feature of Cu-MoS_2_ nanopetals with thinner layers compared with Cu-MoS_2_ nanoboxes provides richer edge sites for Mg^2+^ ions diffusion across the interlayer channels.^50^ As a result, the Mg^2+^ ions diffusion coefficient has been greatly improved in the order of MoS_2_, Cu-MoS_2_ and Cu-MoS_2_@HsGDY. Altogether, in contrast to Cu-MoS_2_ and MoS_2_, an improved rate capability of Cu-MoS_2_@HsGDY with an initial reversible charge capacity of 170.4, 168.4, 157.1 144.2, 130.2, 114.1, and 91 mAh g^–1^ are achieved at 10, 20, 50, 100, 200, 500, and 1000 mA g^–1^, respectively (Figure 6d). And a high charge capacity of 149.7 mAh g^–1^ with an effective recovery of 95% can be maintained when the current density returns to 50 mA g^–1^, which suggests the strong synergistic effect of the hybrid nanocapsules to facilitate the de/intercalation of Mg^2+^ ions. Impressively, when cycled at high charge/discharge rates, it also exhibits high specific capacities of 100 mAh g^–1^ (200 mA g^–1^) and 85.5 mAh g^–1^ (500 mA g^–1^) after 300 cycles (Figure 6e).

It should be noted that a short initial activation process happens on both Cu-MoS_2_ and Cu-MoS_2_@HsGDY but MoS_2_, which could be ascribed to the gradual phase transition of MoS_2_ and the shielding effect of DME solvent upon deep cycling. In details, with the Mg^2+^ intercalation, a certain degree of distortion process happens on the Cu-MoS_2_ nanolayers, which further activate the phase transition from semiconductive 2H-MoS_2_ to metallic 1T-MoS_2_ phase. Upon deep cycling, the proportion of 1T-MoS_2_ from surface to bulk increases gradually, which contributes to the improved capacity by increasing the ion and electron conductivity of the cathode material.^10^ Besides, it has been reported that the shielding effect of DME molecules could decrease the interaction energy barrier between the inserted Mg^2+^ ions and the MoS_2_ lattice, which is supposed to be another reason for the activation process.^10^ To draw a distinction between the diffusion kinetic process and the surface capacitive behavior involved in the Mg^2+^ ion storage process, CV curves of Cu-MoS_2_@HsGDY are recorded at various scan rates (Figure S28).^11,52,53^ As a result, the surface-capacitive kinetics dominate the hierarchical Cu-MoS_2_@HsGDY nanocapsule (e.g., capacitive contribution covers 71.5% at 1.0 mV s^–1^, Figure S28). This electrochemical behavior is consistent with the fluffy structure of Cu-MoS_2_ with both an enlarged interlayer distance and a dominant active Cu–Mo–S phase in basal planes, which provides abundant accessible sites and lower energy barrier for Mg^2+^ ion storage. Consequently, by integrating the merits of electroactive Cu-MoS_2_, inner added skeletons and electron/ion conductive HsGDY, a decent cathode material of Cu-MoS_2_@HsGDY nanocapsule that simultaneously delivers high specific capacity, long cycling stability, and superior rate capability for RMB has been successfully achieved.

## Conclusions

In conclusion, nanostructural engineering of 2D MoS_2_ by a dual-template method with a continuous-conversion mode has been developed. Specifically, we fabricated a Cu-MoS_2_@HsGDY nanocapsule in which both electronic structure modulation and hierarchical nanostructure construction of MoS_2_ are achieved in one process. As a result, the Cu-MoS_2_@HsGDY nanocapsule provides a high-capacity, high rate, and stable cathode material for rechargeable magnesium batteries. On the one hand, the extended π-conjugated structure of HsGDY qualifies it as a conductive additive for Cu-MoS_2_ nanopetals to improve their conductivity, while its hierarchical porous environment favors ion diffusion across the whole material. On the other, the rigid HsGDY and the inner added nanoboxes serve as space-confined capsule and built-in buffers, respectively, to rationally accommodate the volume change during cycling. We believe that the dual-template method reported here enables the engineering of hierarchical nanocomposites with merits of both well-organized geometries and carefully designed functionalities to meet the ongoing challenges for post-lithium-ion battery technologies.^54−57^

## Methods and Materials

### Synthesis of Cu-cysteine@HsGDY Nanowires

Cu-cysteine hybrid nanowires were fabricated according to a literature method.^37^ Briefly, 0.54 mL of ethanolamine was added into 300 mL of deionized water containing 0.723 g of Cu(NO_3_)_2_·6H_2_O and 0.36 g of l-cysteine. After vigorous stirring at room temperature for 1.5 h, the sky blue floccules, denoted as Cu-cysteine, were separated by centrifugation. After drying under vacuum, 800 mg of Cu-cysteine nanowires were dispersed into a 250 mL round-bottomed flask containing tetrahydrofuran (40 mL) and trimethylamine (80 mL) with catalysts of Pd(PPh_3_)_2_Cl_2_ (33.6 mg) and CuI (8.8 mg). Then, 80 mg of 1,3,5-triethynylbenzene was added under argon atmosphere. After stirring at 60 °C for 24 h, the Cu-cysteine@HsGDY core–shell nanowires with a dark yellow color was obtained by washed with ethanol/water mixture for several times.

### Synthesis of Cu-MoS_2_@HsGDY Nanocapsules

The as-prepared Cu-cysteine@HsGDY nanowires were acted as a kind of dual template to evolve the hierarchical porous nanocapsule with multiple skeleton geometries. In details, 10 mg of Cu-cysteine@HsGDY nanowires was dispersed in a 50 mL autoclave containing 10 mL of DMF and 10 mg of (NH_4_)_2_MoS_4_. The autoclave was then subjected to a solvothermal process for 15 h at 210 °C, by the end of which the hierarchical Cu-MoS_2_@HsGDY nanocapsules with a black color were acquired by centrifugation with water and ethanol for several times, respectively.

### Characterization

The SEM images were captured with a Zeiss Merlin SEM. TEM samples were prepared with nickel grids and then on a ThermoFisher Scientific’s Titan ST equipped with a Gatan Image Filter (GIF) Tridiem. TEM tomography was carried out on a Titan ST (FEI Company) operating at 300 kV equipped with a 4000 × 4000 charge-coupled device (CCD) camera (Gatan). The tilt series for tomography reconstruction were acquired by using Xplore 3D tomography software (FEI Company). In this process, the tilt series were captured from −65 to +65° at 2° initial intervals following a Saxton scheme. The tomograms were produced using a back projection algorithm as implemented in the IMOD software. The 3D construction was generated with the segmentation tools implemented in Avizo Fire 8.0 software. The XRD patterns were collected on a Bruker D8 ADVANCE Diffraction System with a Cu Kα irradiation (λ= 1.5406 Å). The Raman spectrum was recorded with a Horiba Aramis with a laser wavelength of 473 nm excitation. Nitrogen sorption measurement was taken with a Micromeeitics-TriStar II system. The surface area and the pore size distributions were calculated using the Brunauer–Emmett–Teller method and density functional theory (DFT), respectively. The FTIR spectra were conducted on a FTIR-is10 spectrometer with a diamond. The ICP-AES was analyzed by Varian 720-ES spectrometer. The XPS study was taken with the Axis Ultra instrument (Kratos Analytical, vacuum < 10^–9^ mbar) equipped with a monochromatic Al Kα X-ray (*h*υ = 1486.6 eV) source carried out at 150 W. The data was analyzed with the commercially available software of Casa-XPS. The solid ^13^C NMR spectra were carried out on the WB Bruker 600 AVANAC III spectrometer equipped with a 2.5 mm double resonance MAS Bruker Probe (BrukerBioSpin, Rheinstetten, Germany). Bruker Topspin 3.2 software (Bruker BioSpin, Rheinstetten, Germany) was used to collect and analyze the data. For studying the composition and morphology evolution of the Cu-MoS_2_@HsGDY electrode after cycling, the cycled batteries were disassembled in a glovebox, and the electrodes were rinsed several times with dimethyl carbonate (DMC). After drying in vacuum for 30 min, the electrodes were transferred to conduct XRD, XPS, and TEM characterizations.

### Electrochemical Performance for RMB

A typical kind of CR2032 (MTI, Inc.) coin-type cell was assembled to evaluate their RMB performance. The working electrodes were prepared by mixing 70 wt % active materials (Cu-MoS_2_@HsGDY or Cu-MoS_2_ or MoS_2_ or HsGDY) with 20 wt % acetylene black (MTI, Inc.), and 10 wt % poly(vinylidene fluoride) (PVDF, MTI Inc.) in *N*-methyl-2-pyrrolidone (NMP, MTI) and fully grinding the mixture. Afterward, the slurry was uniformly coated on a piece of molybdenum foil current collector and dried in vacuum at 80 °C over 24 h. Then it was punched into disks with a dimeter of ∼14 mm with an active material mass loading around 2 mg cm^–2^. For electrochemical measurement, the Mg metal foil was utilized as the counter electrode and reference electrode, Whatman glass fibers served as the separator, and a 0.25 M solution of MgCl_2_ and AlCl_3_ (1:2 mol ratio) in 1,2-dimethoxyethane (DME) was used as the electrolyte. The electrochemical performance of these assembled cells was carried out on a NEWWARE battery test system in the voltage window from 0.1 to 2.2 V vs Mg/Mg^2+^. The cyclic voltammetry and electrochemical impedance measurements were carried out on a BioLogic VMP3 electrochemical workstation. All the specific capacities and current densities were calculated on the basis of the mass of the active material.

## Supporting Information Available

SEM, TEM, XRD, FTIR, BET, XPS, EIS Nyquist plots and solid ^13^C NMR spectra of HsGDY, Cu-cysteine@HsGDY, CuS@HsGDY and Cu-MoS_2_@HsGDY, respectively; 3D electron tomography of Cu-MoS_2_@HsGDY; *ex situ* TEM, XRD and XPS spectra of Cu-MoS_2_@HsGDY upon cycling (PDF)
Videos S1 and S2: TEM videos of Cu-MoS_2_@HsGDY nanocapsule under 2D microscopy and 3D tomography, respectively (AVI, AVI)

## Supplementary Material

nn1c09405_si_001.pdf

nn1c09405_si_002.avi

nn1c09405_si_003.avi
