# Supplementary material for: Hierarchical Nanocapsules of Cu-Doped MoS2@H-Substituted Graphdiyne for Magnesium Storage
Source: ACS Nano. 2022 Mar 7;16(3):3955–64. doi: 10.1021/acsnano.1c09405 (PMC8945386; doi:10.1021/acsnano.1c09405)
Supplement: Supplementary file 1 — nn1c09405_si_001.pdf [file nn1c09405_si_001.pdf]

Supporting Information

Hierarchical Nanocapsules of Cu-Doped MoS_2@H-Substituted Graphdiyne for Magnesium Storage

Sifei Zhuo,[†,‡,*] Gang Huang,[‡] Rachid Sougrat,[§] Jing Guo,[‡] Nini Wei,[§] Le Shi,[‖] Renyuan Li,[‖] Hanfeng Liang,[‡] Yusuf Shi,[‖] Qiuyu Zhang,[†] Peng Wang,[‖,Δ,*] and Husam N. Alshareef[‡,*]

[†]School of Chemistry and Chemical Engineering, Xi'an Key Laboratory of Functional Organic Porous Materials, Northwestern Polytechnical University, Xi'an 710072, PR China

[‡]Materials Science and Engineering, [‖]Water Desalination and Reuse Center, Biological and Environmental Science and Engineering Division, and [§]Core Labs, King Abdullah University of Science and Technology (KAUST), Thuwal 23955-6900, Saudi Arabia

[Δ]Department of Civil and Environmental Engineering, The Hong Kong Polytechnic University, Hong Kong, PR China.

E-mail: sifei.zhuo@nwpu.edu.cn; peng.wang@kaust.edu.sa; husam.alshareef@kaust.edu.sa

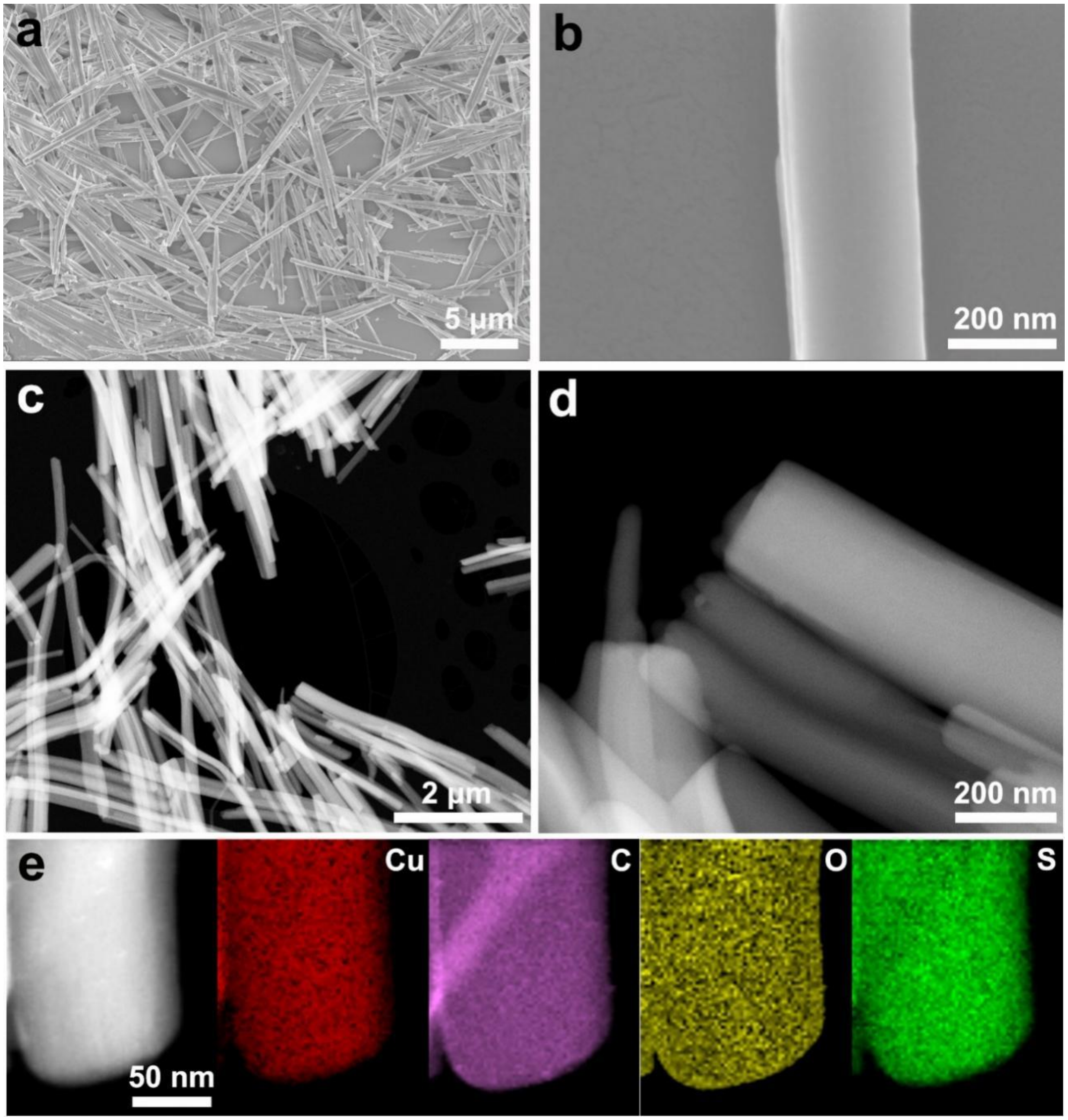

Figure S1. (a,b) SEM images, (c,d) HAADF-STEM images, (e) STEM-EELS elemental mapping images of the Cu-cysteine nanowires. SEM and TEM images indicate that these high-yield products were solid nanowires with diameter around 200 nm and length up to 10 µm. The uniform distribution of elemental of Cu, C, O and S shown in the STEM-EELS mapping verified the homogeneous coordination of Cu^{2+} with L-cysteine.

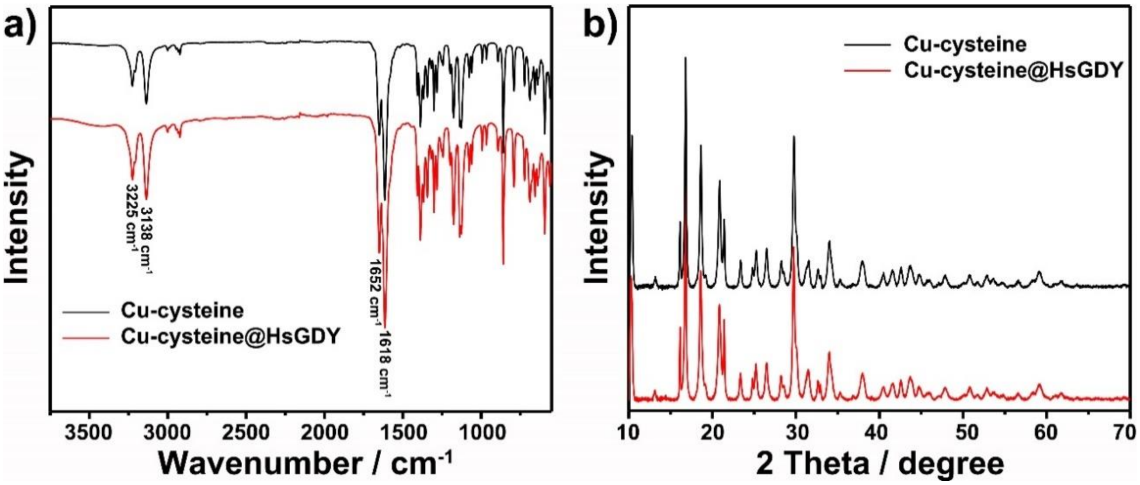

Figure S2. (a) FTIR spectra, (b) XRD patterns of the synthesized Cu-cysteine and Cu-cysteine@HsGDY nanowires with characteristic vibration peaks. In details, the peaks centred at 3225 cm^{-1} and 3138 cm^{-1} belong to the amido group (-NH_2). The peaks located at 1652 cm^{-1} and 1618 cm^{-1} are assigned to vibrations of acylamino group (−CO−NH_2−).[1] All of these results demonstrate that Cu^{2+} have been successfully coordinated with L-cysteine into a 1D nanowires. And the consistent FTIR spectra and XRD patterns before and after HsGDY coating suggest that the copolymerization process does not have any influence on the Cu-cysteine precursor.

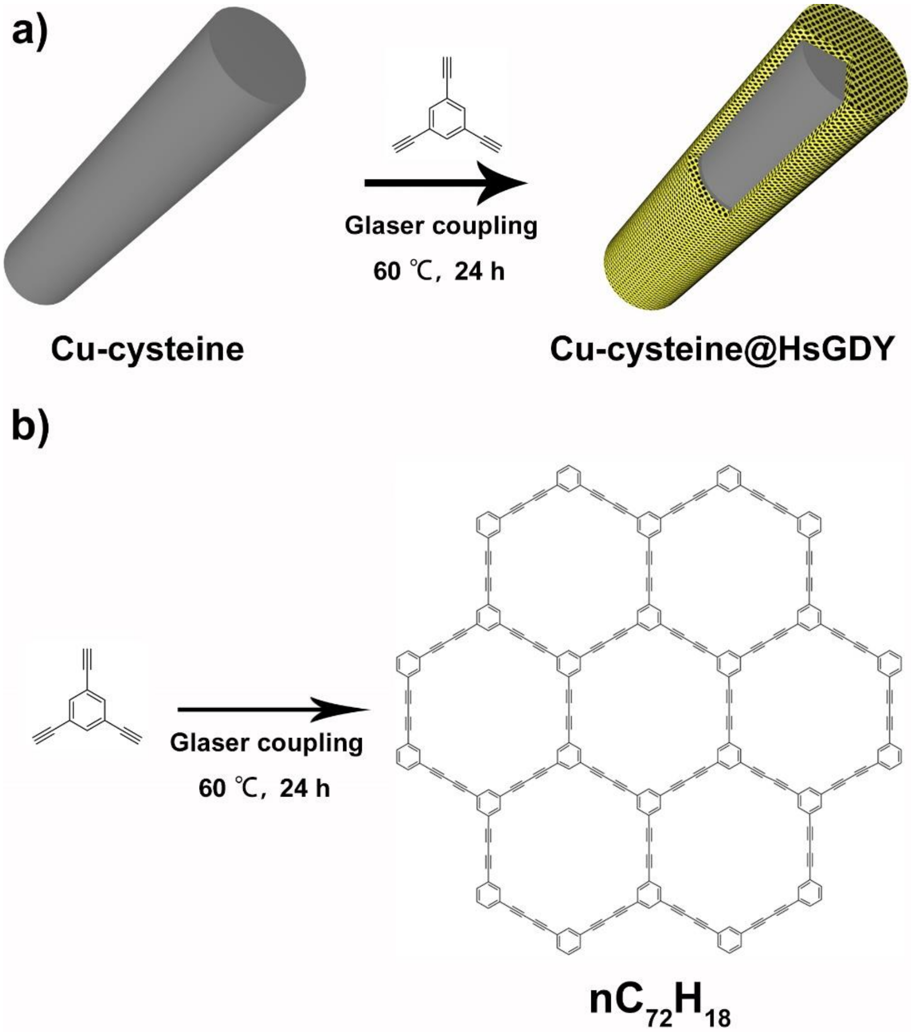

Figure S3. (a) Schematic illustration of the fabrication of Cu-cysteine@HsGDY by coating Cu-cysteine with HsGDY through Glaser coupling reaction of 1,3,5-triethynylbenzene; (b) Schematic synthesis of HsGDY microporous networks. The predictability and controllability of HsGDY at low temperature (60 ℃) distinguish it from hard carbon. By virtue of these merits, we have developed a novel dual-template method that makes use of HsGDY nanolayer to confine a self-template of Cu-cysteine with controlled thickness. Since the conformal coating layer of HsGDY is formed by an *in situ* Glaser coupling reaction of 1,3,5- triethynylbenzene, it is proposed that the controllable HsGDY nanolayers could be easily crosslinked on many kinds of nanostructures under low temperature. In addition, the selection of HsGDY in this synthesis is

judicious as it physically confines the sacrificial template in place and allows mass transfer in and out through its micropores to permit the *in situ* chemical transformation reaction, which further distinguish it from carbon.

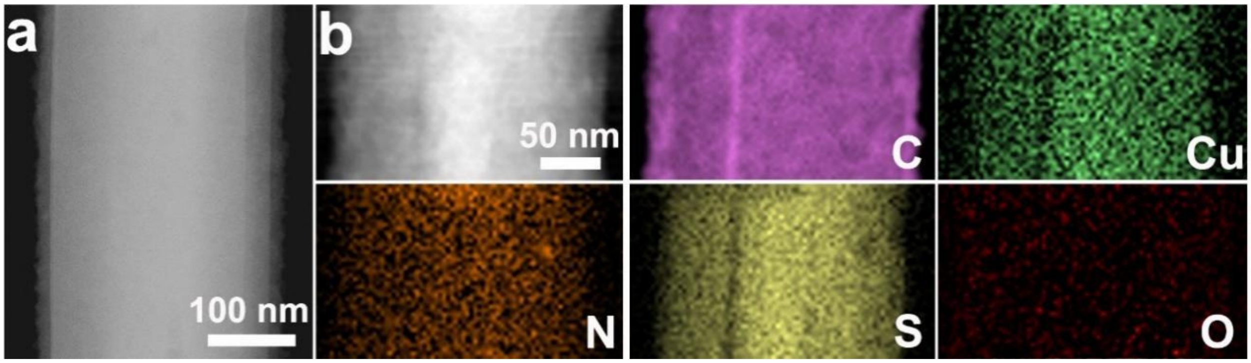

Figure S4. (a) HAADF-STEM image and (b) STEM-EELS elemental mappings of the as-prepared hybrid Cu-cysteine@HsGDY nanowires. The STEM-EELS elemental mappings indicate the homogeneous distribution of elemental Cu, S, O and N encapsulated by a hetero-epitaxial HsGDY layer that rich in carbon.

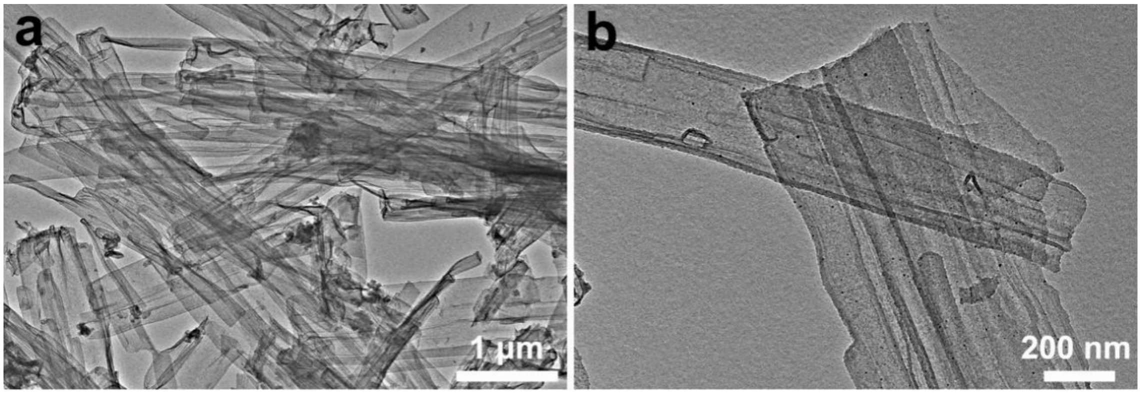

Figure S5. (a,b) TEM images of the HsGDY nanotubes, which is fabricated by acid etching the Cu-cysteine cores of Cu-cysteine@HsGDY hybrid nanowires.

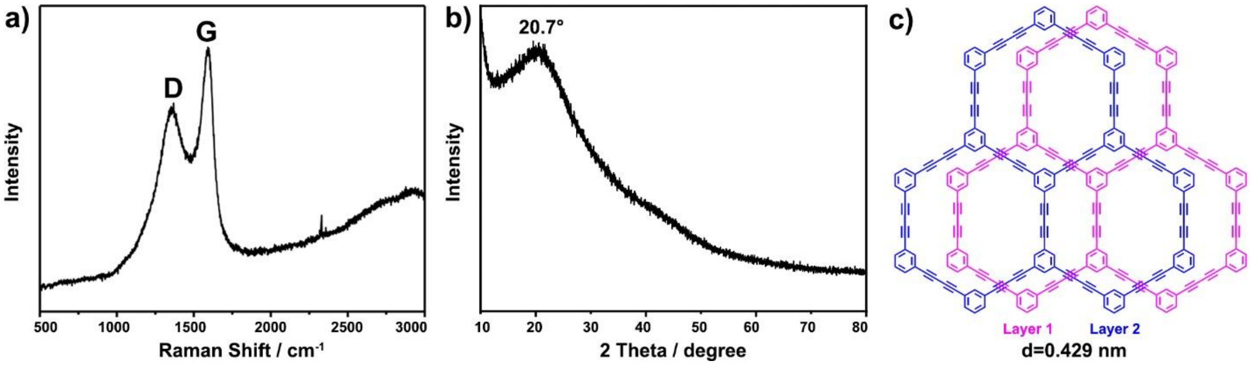

Figure S6. (a) Raman spectrum, (b) XRD pattern and (c) layered structure of hydrogen-substituted graphdiyne (HsGDY). The peak located at 20.7° for HsGDY can be corresponded to the interlayer spacing of 4.29 Å, which is similar with the reported value by Yuliang Li.[2] Although the layered structure of HsGDY framework is confirmed, its crystallinity is poor. The major obstacle for the crystallinity lies in the fact of the conformational fluctuation of HsGDY during the Glaser-coupling reaction process.[2,3] In details, during the polymerization process, the free rotation around alkyne-aryl single bonds of the monomers results in a highly branched framework of HsGDY with poor ordering instead of a crystalline layer-by-layer planar framework. Having said that, we can precisely control the apparent thickness of HsGDY from several to tens of nanometer. In this work, the apparent thickness value of the HsGDY capsule is set to around 10 nm. For one reason, it is strong enough to qualify HsGDY withstand the volume change both happened in the chemical transformation procedure and the reversible Mg^{2+} ions intercalation/deintercalation process. For another, it is sufficient to work as an effective electron/ion channel to improve the kinetics of the cathode.

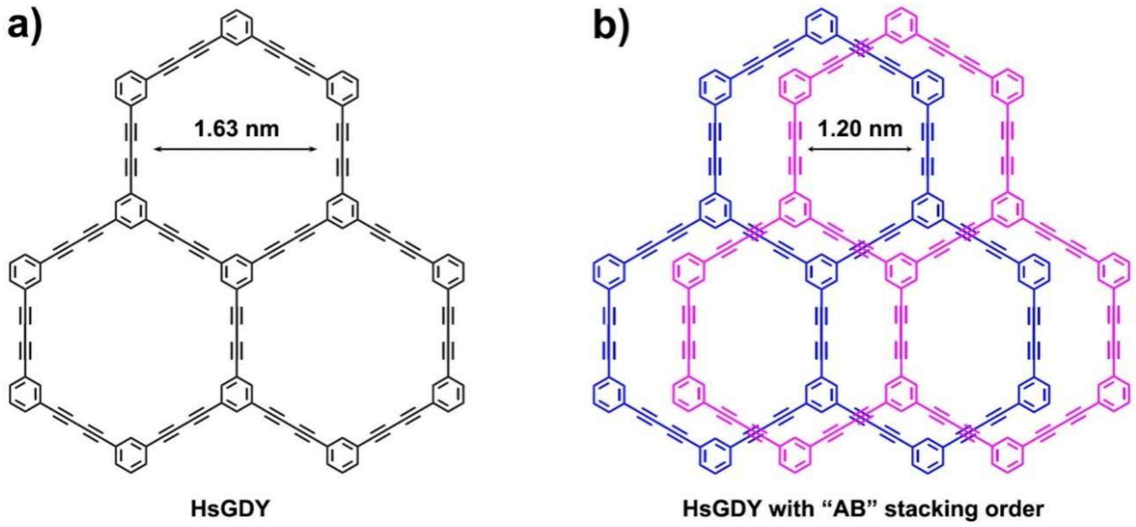

Figure S7. (a) Chemical structure of single layered HsGDY, and (b) 2D layered HsGDY with “AB” stacking mode. According to the chemical structure of HsGDY consisting of benzene rings and butadiyne linkages, the theoretical pore size of single layered HsGDY is 1.63 nm. However, the experimental value of the fabricated HsGDY nanocapsule is calculated to be around 1.2 nm. The difference between theoretical and experimental values suggests the AB stack mode of HsGDY layer.

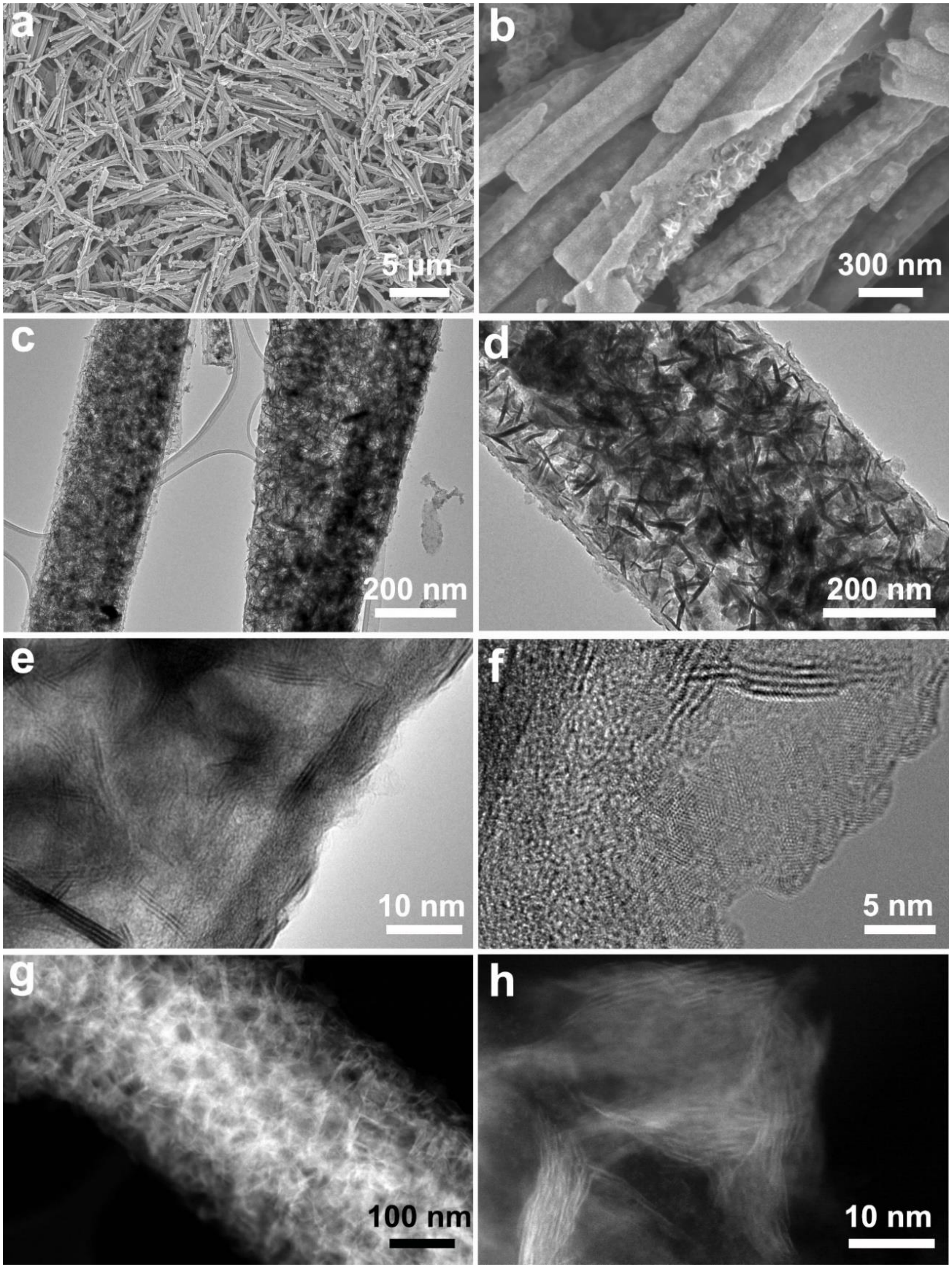

Figure S8. (a,b) SEM images, (c-f) TEM images and (g,h) STEM images of the as-prepared Cu-MoS_2@HsGDY nanocapsules.

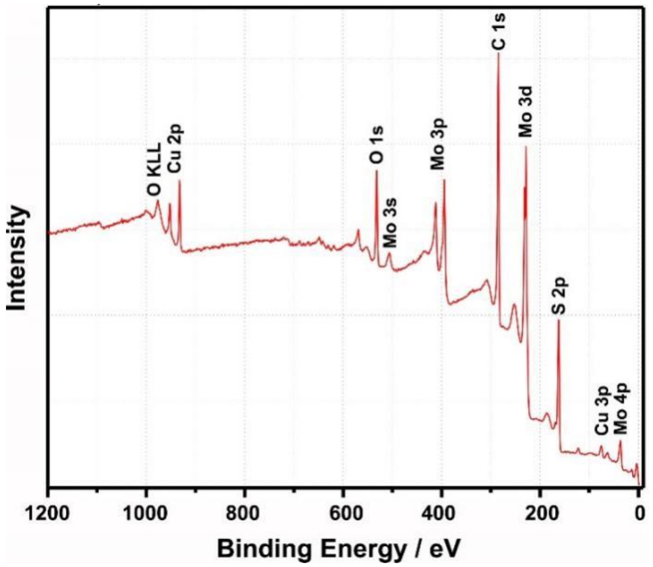

Figure S9. XPS spectrum of the as-prepared Cu-MoS_2@HsGDY nanocapsules. It clearly identifies the existence of Cu, Mo, S and C.

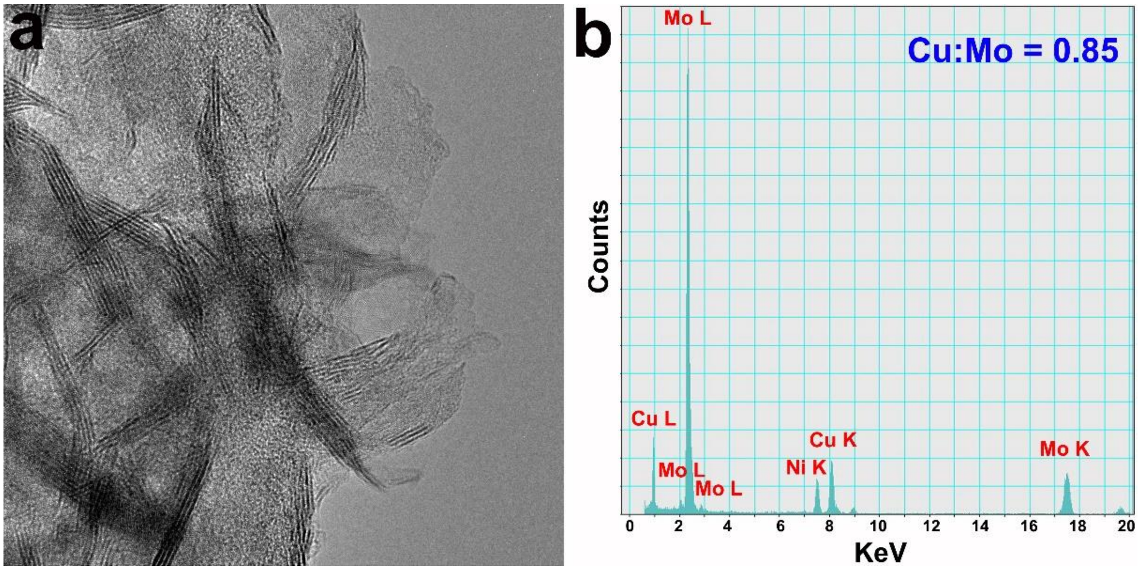

Figure S10. (a) HRTEM image and (b) the corresponding EDS spectrum of the as-prepared Cu-MoS_2@HsGDY nanocapsule. As no CuS phase is detected in the HRTEM image, these nanopetals can be identified as of Cu-MoS_2. As shown in Figure S10b, the atomic ratio of Cu/Mo is around 0.83. According to the collision theory in chemical kinetics, the ion concentration plays a key role in the successful bond formation. Herein, since the nucleation process of MoS_2 happens on the surface of CuS nanocubes, where is rich in Cu^{2+}, vigorous collisions take place and accelerates the formation of cross-linked Cu-MoS_2 nanopetals with dominated Cu-Mo-S phase. As a result, the distribution of the incorporated Cu in the structure is quite homogeneous and a high content of Cu is achieved.

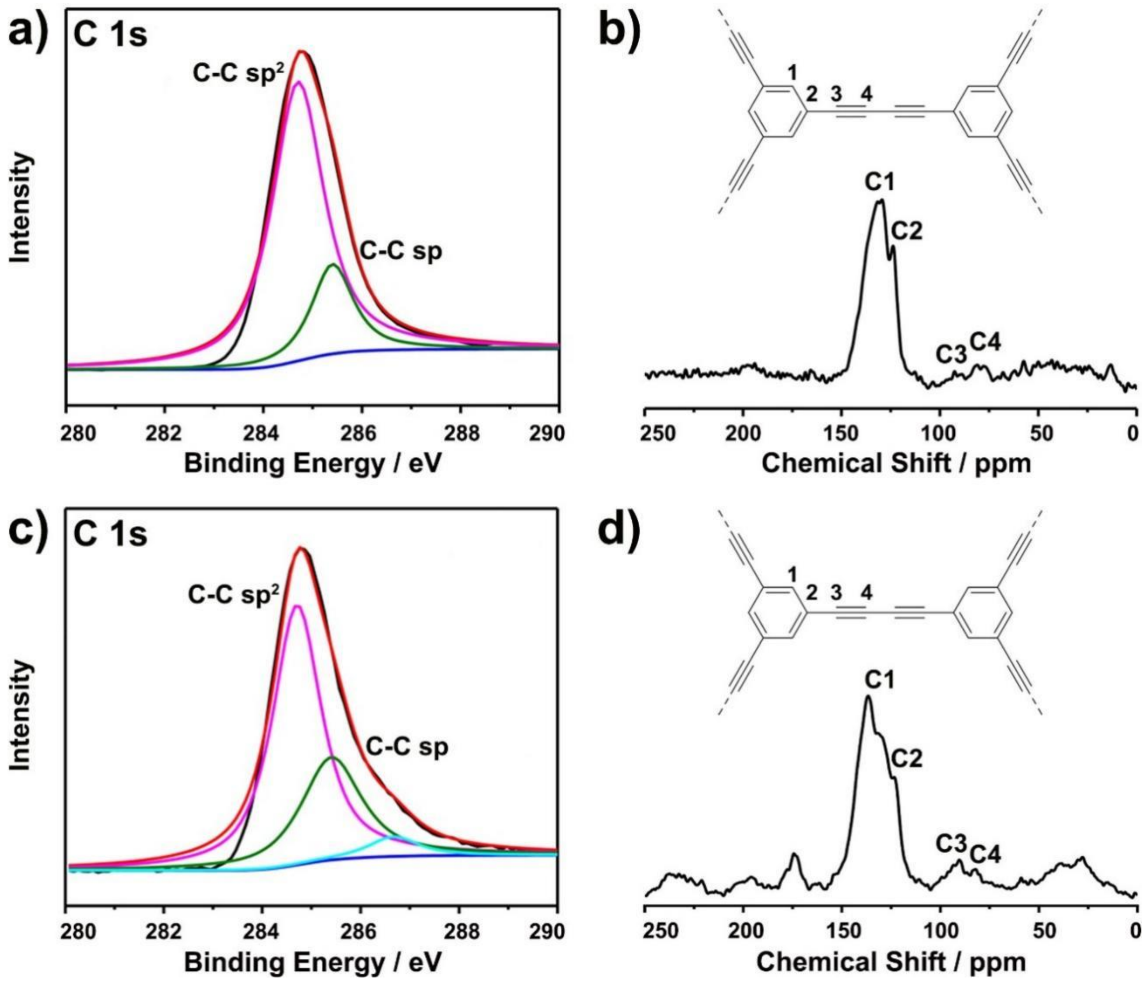

Figure S11. (a,c) XPS and (b,d) Solid ^{13}C NMR spectra of hydrogen-substituted graphdiyne before (a,b) and after (c,d) chemical transformation. The consistent spectra of both XPS and Solid ^{13}C NMR indicate that the chemical structure of HsGDY is left intact, which qualify it as functional support for Cu-MoS_2 for magnesium storage.

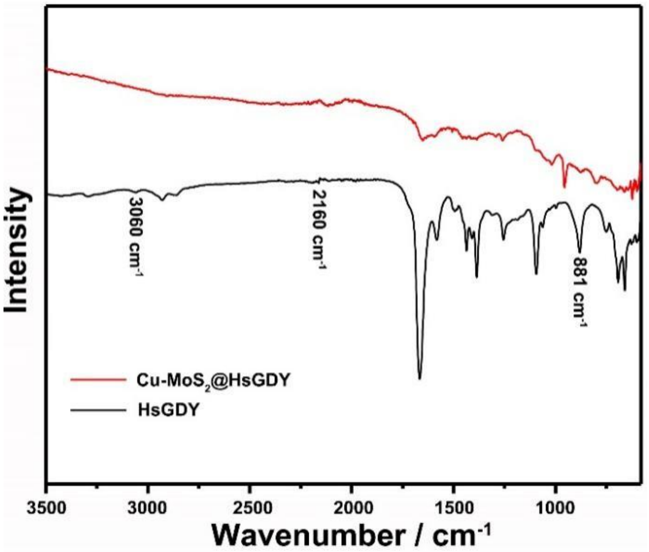

Figure S12. FTIR spectra of the HsGDY nanotubes and the as-prepared Cu-MoS_2@HsGDY nanocapsules. The vibration peaks shown at 2160 cm^{-1} for C≡C bond, and 881 cm^{-1}, 3060 cm^{-1} for aromatic C-H suggest the structure of HsGDY as poly(phenylene butadiynylene) with sp- and sp^2- hybridized carbon atoms. The consistent vibration peaks of Cu-MoS_2@HsGDY compared with HsGDY verify that the chemical structure of HsGDY remain unchanged after solvothermal treatment.

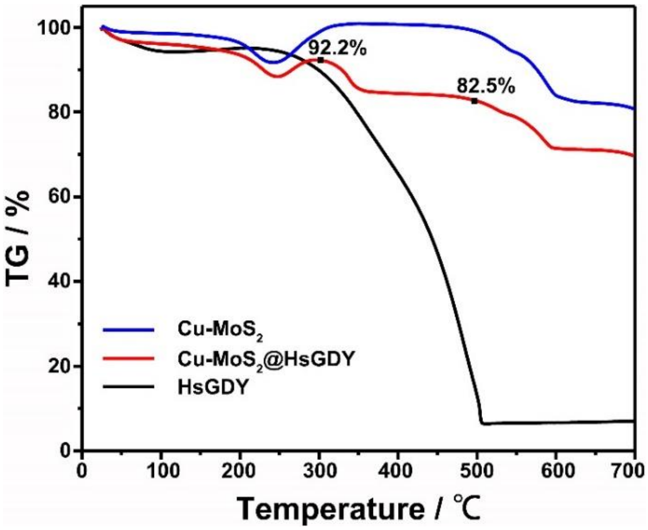

Figure S13. TGA profiles of the as-prepared Cu-MoS_2, HsGDY and Cu-MoS_2@HsGDY. The weight percentage of HsGDY in the capsule of Cu-MoS_2@HsGDY is estimated to be 10 wt %.

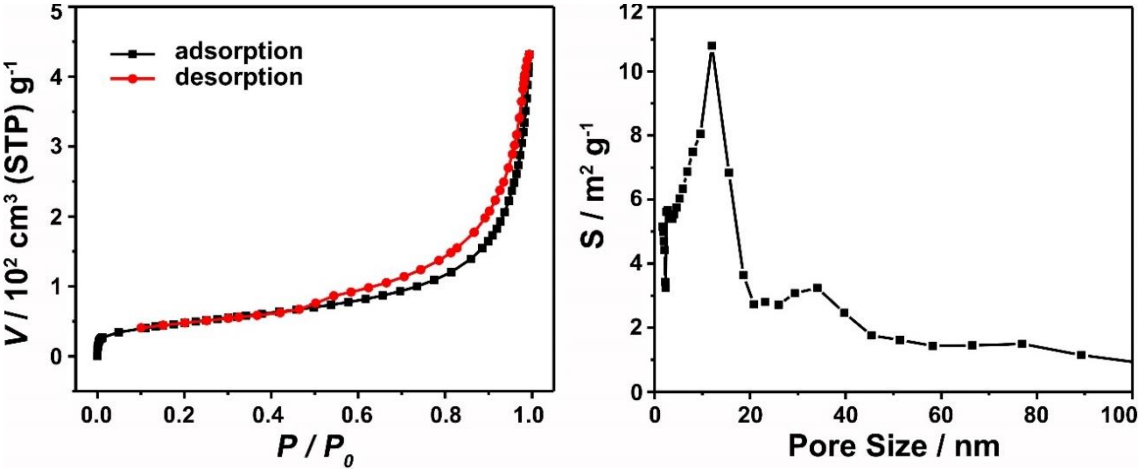

Figure S14. (a) N_2 adsorption/desorption isotherms of the as-prepared Cu-MoS_2@HsGDY nanocapsule, and (b) the corresponding pore size distribution analysed by Barrett-Joyner-Halenda (BJH) method. In addition to micropores of HsGDY, there are also two kinds of mesopores exist in these Cu-MoS_2@HsGDY nanocapsules, which are located at 12 nm and 34 nm, respectively.

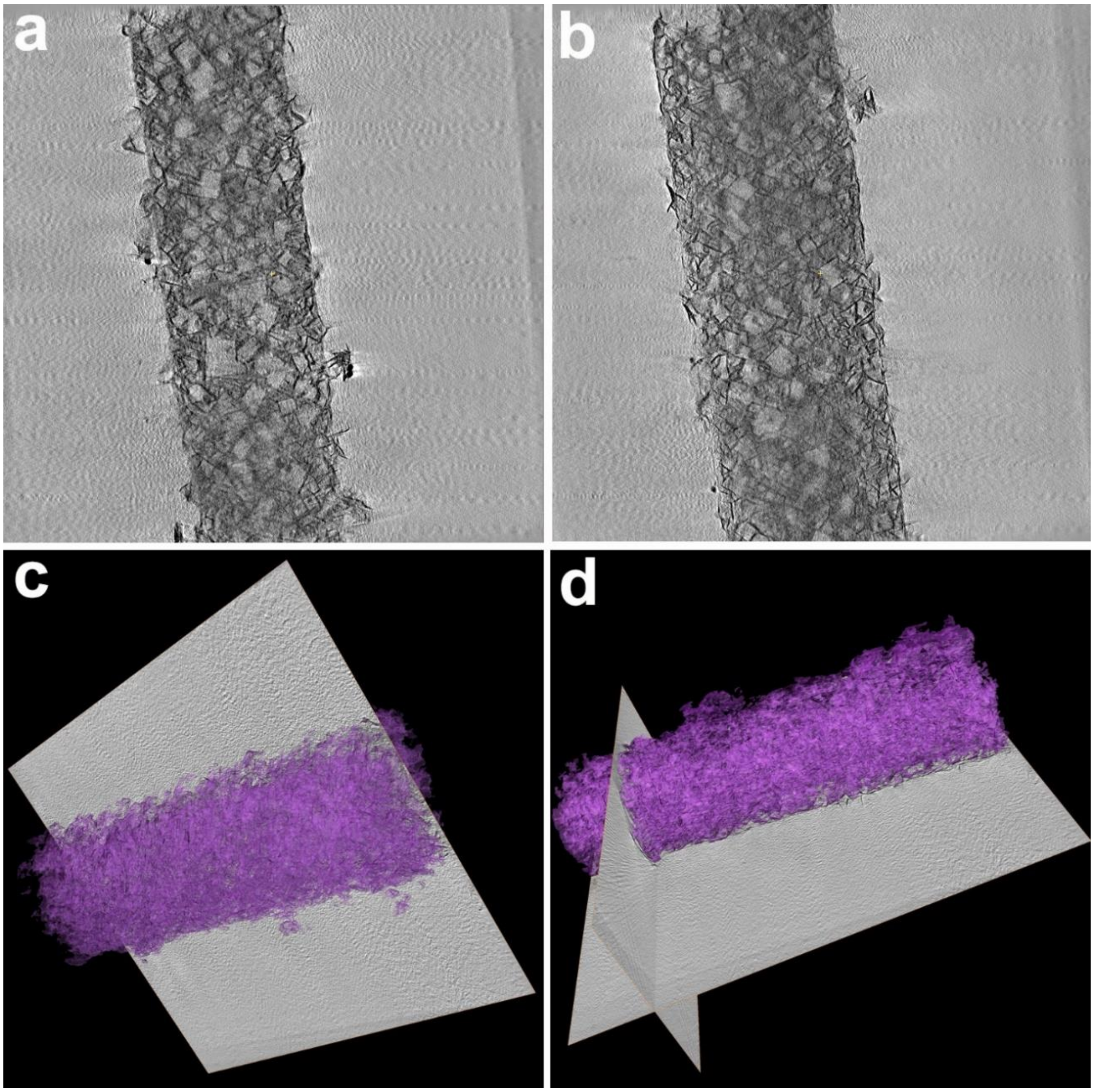

Figure S15. (a,b) Typical virtual cross-sections of the as-prepared Cu-MoS_2@HsGDY taken from the original 3D tomogram at different tilt angles ; (c,d) TEM tomography: 3D volume of the Cu-MoS_2@HsGDY.

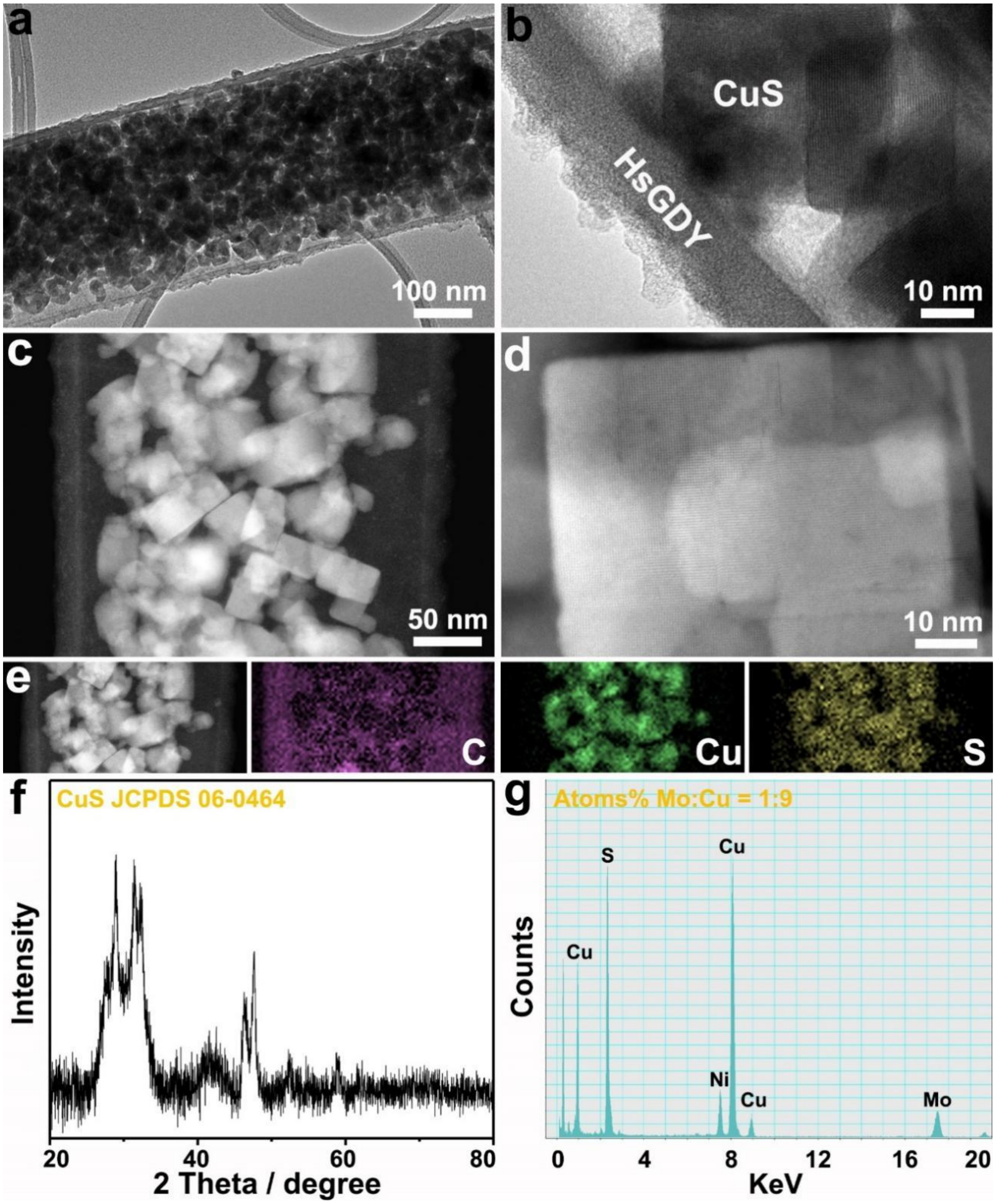

Figure S16. (a,b) TEM images, (c,d) HAADF-STEM images, (e) side-view STEM-EELS elemental mappings, (f) XRD pattern and (g) EDS spectrum of the CuS@HsGDY intermediate template.

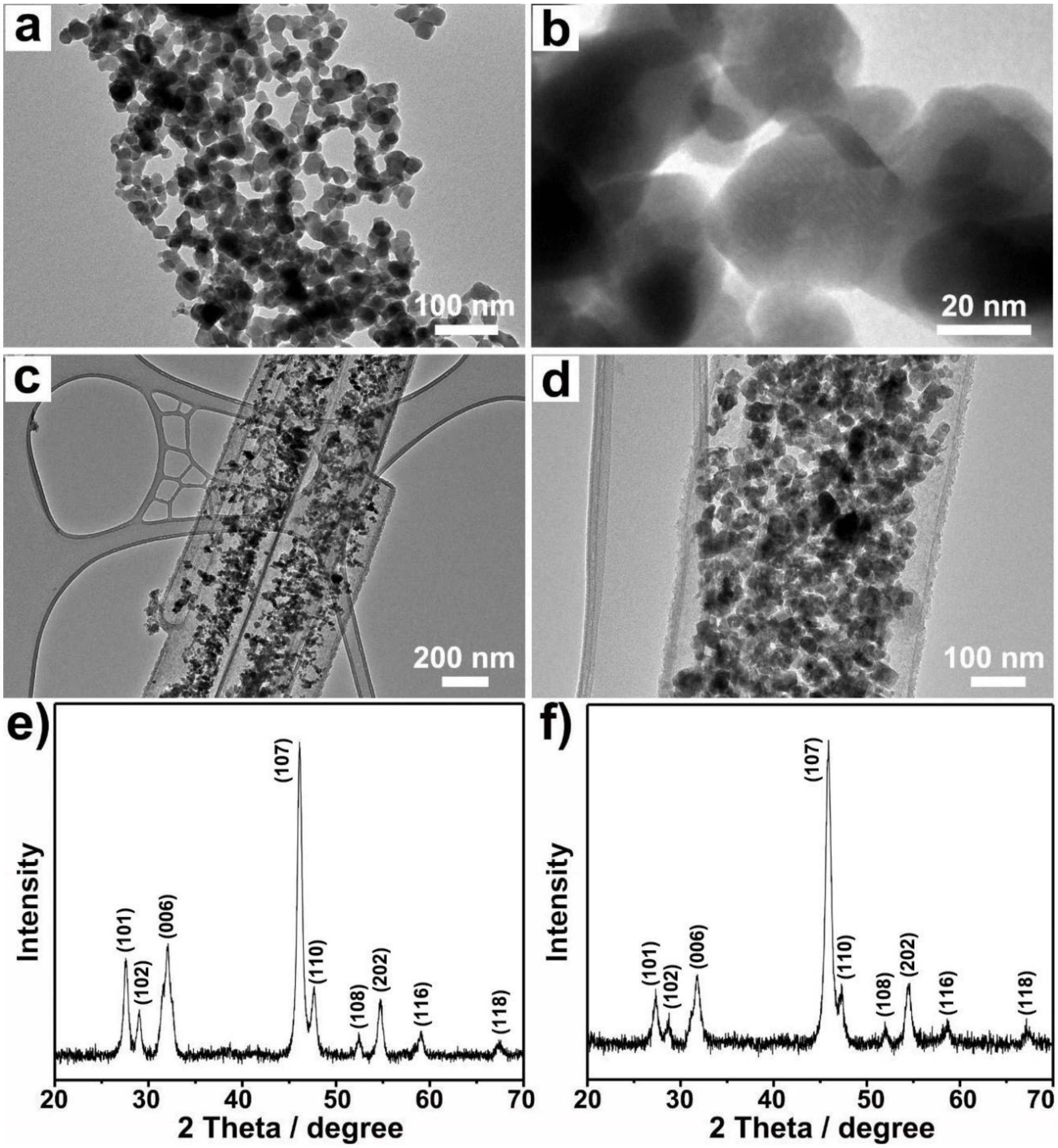

Figure S17. (a-d) TEM images and (e,f) XRD patterns of CuS nanoparticles and CuS@HsGDY nanocapsules derived from thermal decomposition of Cu-cysteine (a,b,e) and Cu-cysteine@HsGDY (c,d,f) in DMF solvent, respectively. Since no additional sulfur source was added during the hydrothermal reaction process, the CuS nanoparticles were definitely derived from the self-decomposition of Cu-cysteine. In other words, the reactivity of L-cysteine makes it *in situ* sulfur source confined in HsGDY capsule.

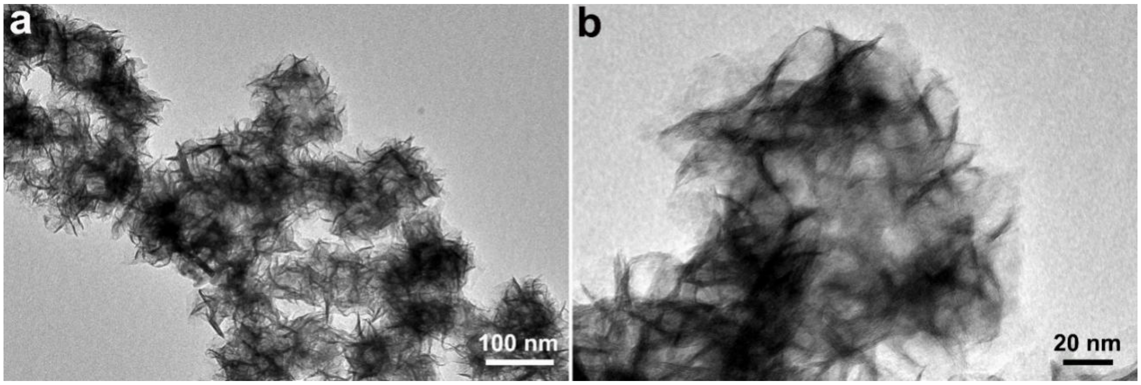

Figure S18. TEM images of the products when we utilize Cu-cysteine nanowires to react with $(NH_4)MoS_4$ under the same conditions. As expect, the nanosheet-based Cu-MoS_2 nanoboxes are fabricated, which further confirms the transformation mechanism via CuS nanocubes. In details, the solid CuS nanocubes derived from the self-decomposition of Cu-cysteine were further act as the secondary self-template to derive the ultimate nanosheet-based Cu-MoS_2 nanoboxes (~100 nm) by reaction with the *ex-situ* $(NH_4)_2MoS_4$.

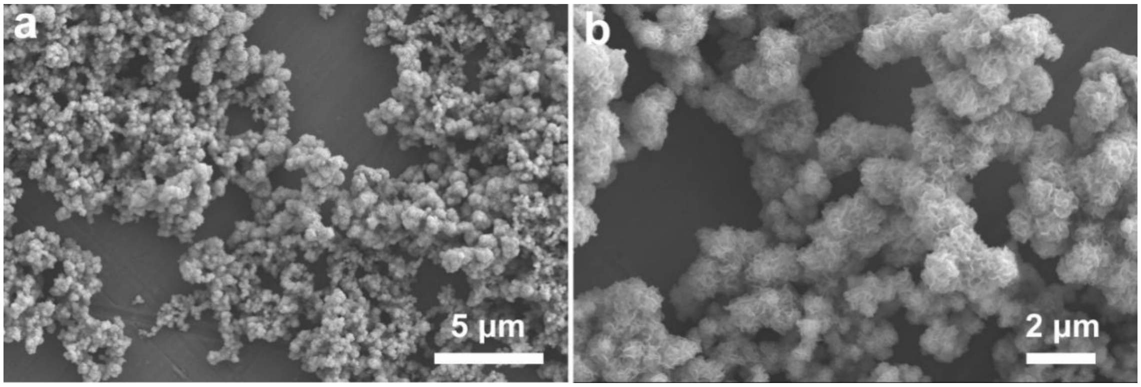

Figure S19. SEM images of the MoS_2 nanospheres fabricated by treatment of $(NH_4)MoS_4$ under the same conditions without any precursors. Without direction of the self-template of CuS nanocubes, nanosheet-based MoS_2 spherical aggregates with a diameter around 1.5 μm are generated.

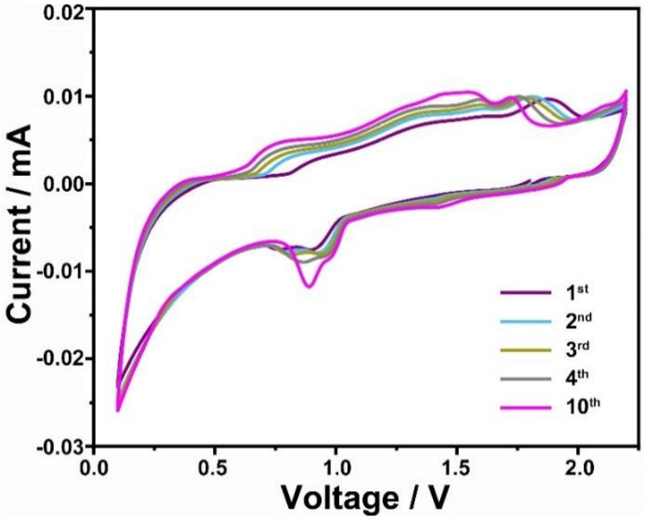

Figure S20. Cyclic voltammetry (CV) curves of Cu-MoS_2@HsGDY at a scan rate of 0.1 mV s^{-1} between 0.1 and 2.2 V. The reduction peak located at 0.9 V *vs* Mg/Mg^{2+} corresponds to the intercalation of Mg^{2+} into the interlayers of Cu-MoS_2, while the oxidation peak at around 1.8 V *vs* Mg/Mg^{2+} is related to the deintercalation of Mg^{2+} from the Cu-MoS_2 interlayers. It is worth noting that the reduction at 0.1 V and oxidation peak at 0.7 V *vs* Mg/Mg^{2+} may be result from the phase transition between 2H-MoS_2 and 1T-MoS_2 upon cycling.

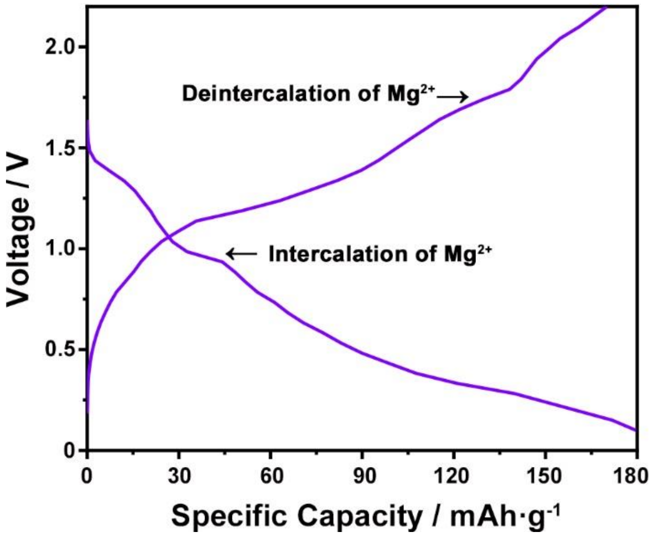

Figure S21. Discharge/charge profiles of Cu-MoS_2@HsGDY nanocapsules at 10 mA g^{-1}. the apparent plateau at around 0.9 V in the discharge curve is assigned to the Mg^{2+} intercalation process, while the plateau at about 1.8 V is corresponded to the Mg^{2+} deintercalation process. These electrochemical behaviours are consistent with the cyclic voltammetry (CV) curves of Cu-MoS_2@HsGDY (Figure S20) with main redox peaks at around 0.9/1.8 V, suggesting the reversible intercalation/deintercalation of Mg^{2+} into Cu-MoS_2 interlayers.

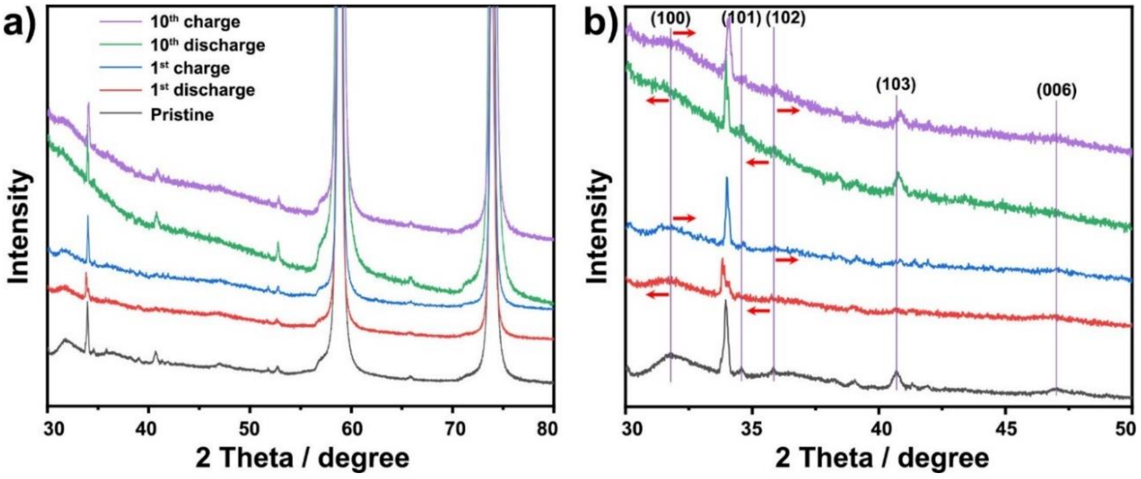

Figure S22. *Ex-situ* XRD patterns of Cu-MoS_2@HsGDY at different discharge/charge stages. The diffraction peaks of Cu-MoS_2@HsGDY are well preserved and no new species are generated either in the charge or discharge states during the initial 10 cycles, which suggests the intercalation mechanism of Cu-MoS_2 without conversion reaction. Yet it is worth noting that the (100) peaks broaden to some extent along with decrease in intensity, which can be ascribed to the formation of nanograins that favorable for reversible Mg^{2+} ions storage. Besides, in the magnified patterns, it should be noted the peaks e.g. (100) and (102) shift towards low-angle and high-angle positions, respectively, in the discharge and charge states. Therefore, these *ex-situ* XRD patterns with crystal lattice expand/contract information suggest the intercalation mechanism of Cu-MoS_2@HsGDY nanocapsules without conversion reaction.

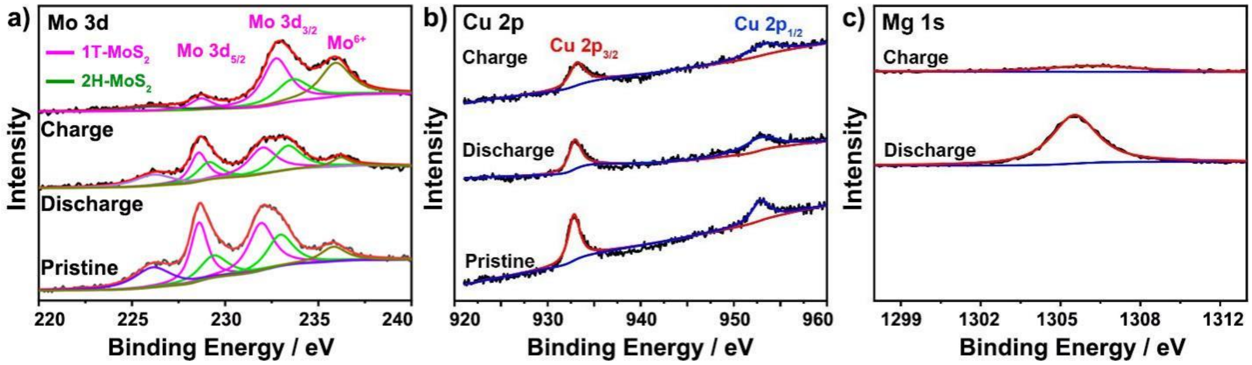

Figure S23. *Ex-situ* XPS spectra of Cu-MoS_2@HsGDY upon cycling. No valence change take place in the doped Cu^{2+} during the discharge process. We assume that there exists a phase transition between 2H and 1T phases of Cu-MoS_2 during the discharge/charge process. A certain degree of distortion process happens on the Cu-MoS_2 nanolayers, which further activate the phase transition from semi-conductive 2H-MoS_2 to metallic 1T-MoS_2 phase. Upon deep cycling, the proportion of 1T-MoS_2 from surface to bulk increases gradually, which contributes to the improved capacity by increasing the ion and electron conductivity of the cathode material. Notably, it has been reported that the additional charges from Mg^{2+} ions will be transferred to MoS_2 to stabilizes the 1T structure. Therefore, in the charged state, although the top surface layer of Cu-MoS_2 possesses a high content of 1T-MoS_2 phase, the majority of the Cu-MoS_2 underneath the surface has been transferred back to the 2H-MoS_2 structure.

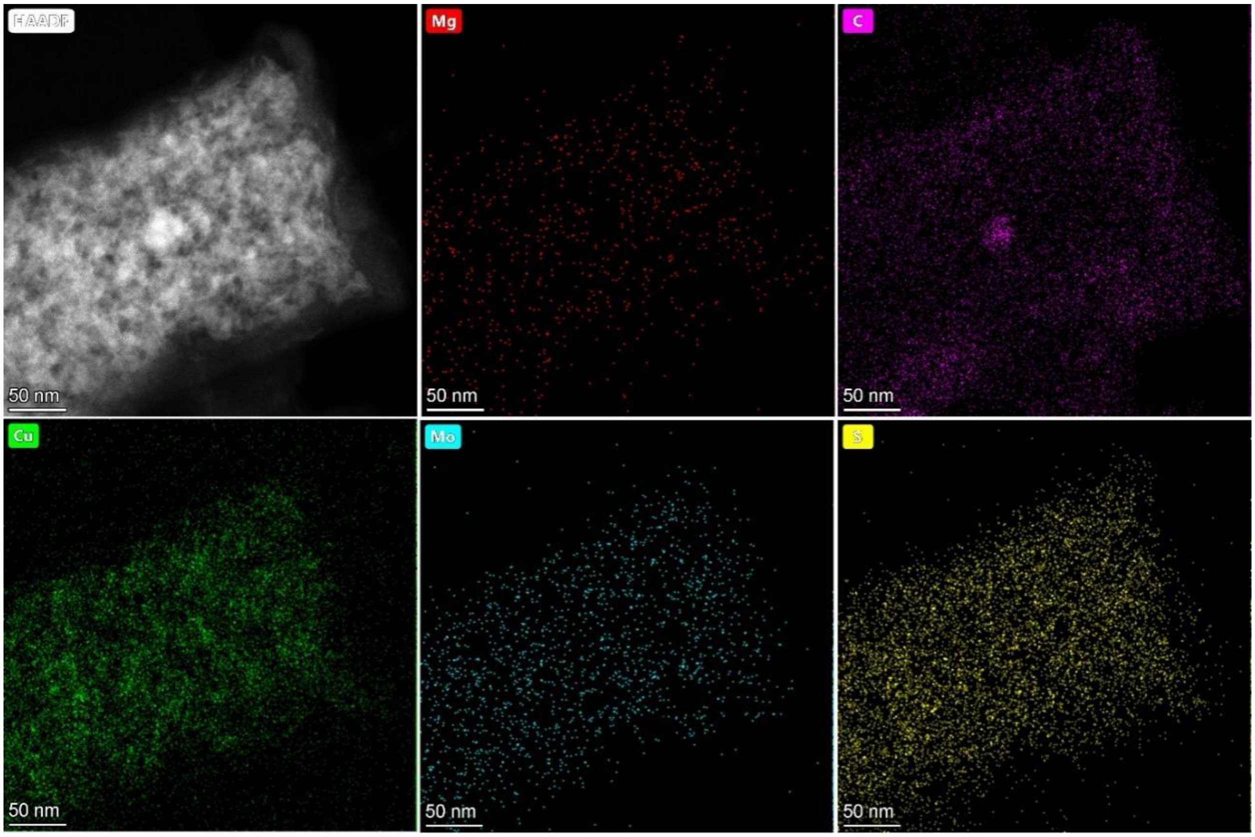

Figure S24. TEM-EDS mapping of the Cu-MoS_2@HsGDY nanocapsules in the discharging state. As expected, the homogenous distribution of Mg along with Cu, Mo, and S evidently affirms the successful intercalation of Mg^{2+} in Cu-MoS_2.

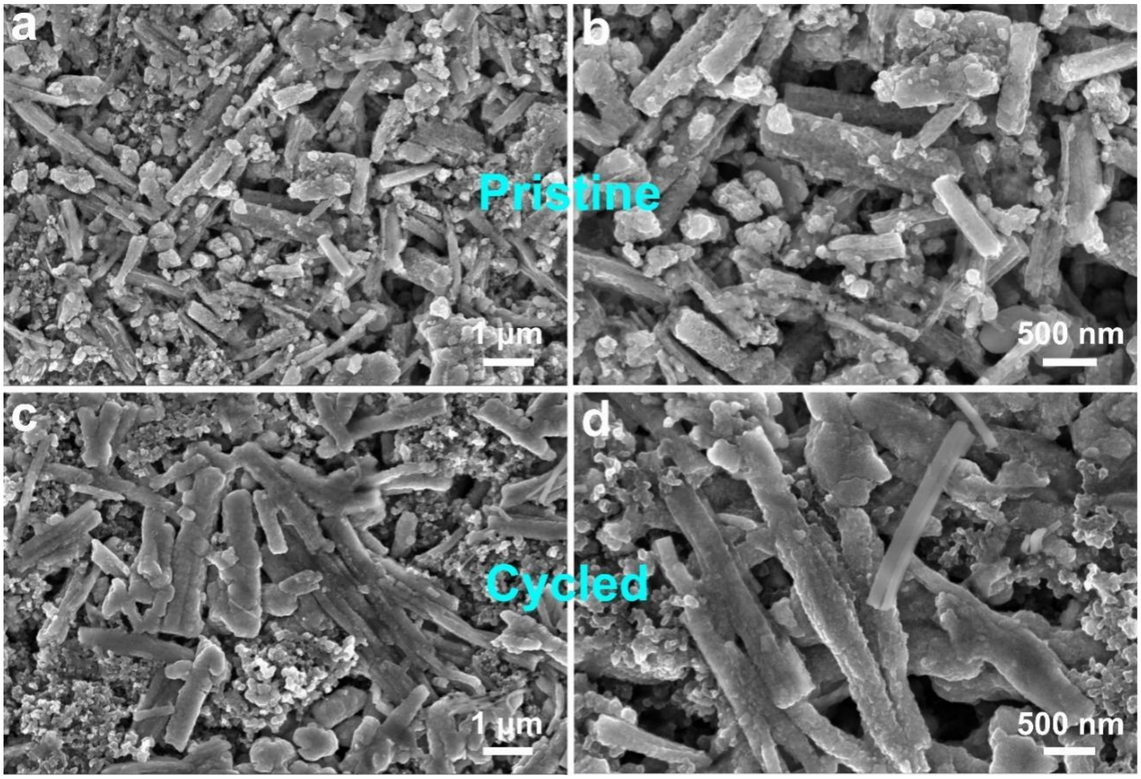

Figure S25. SEM images of Cu-MoS_2@HsGDY before (a,b) and after (c,d) 200 cycles at 50 mA g^{-1}.

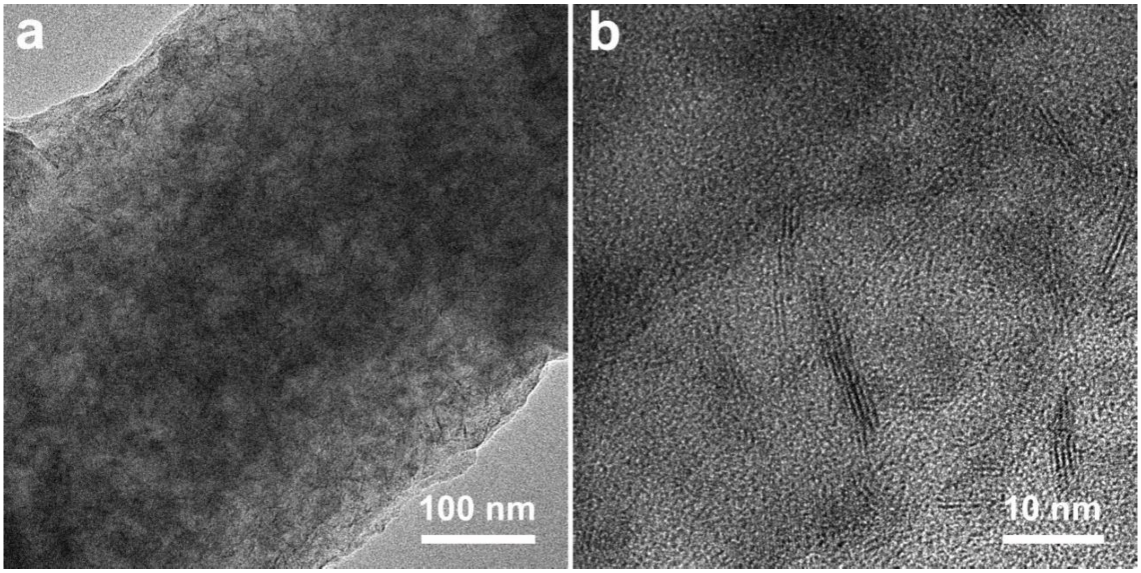

Figure S26. TEM images of Cu-MoS_2@HsGDY after 200 cycles at 50 mA g^{-1}

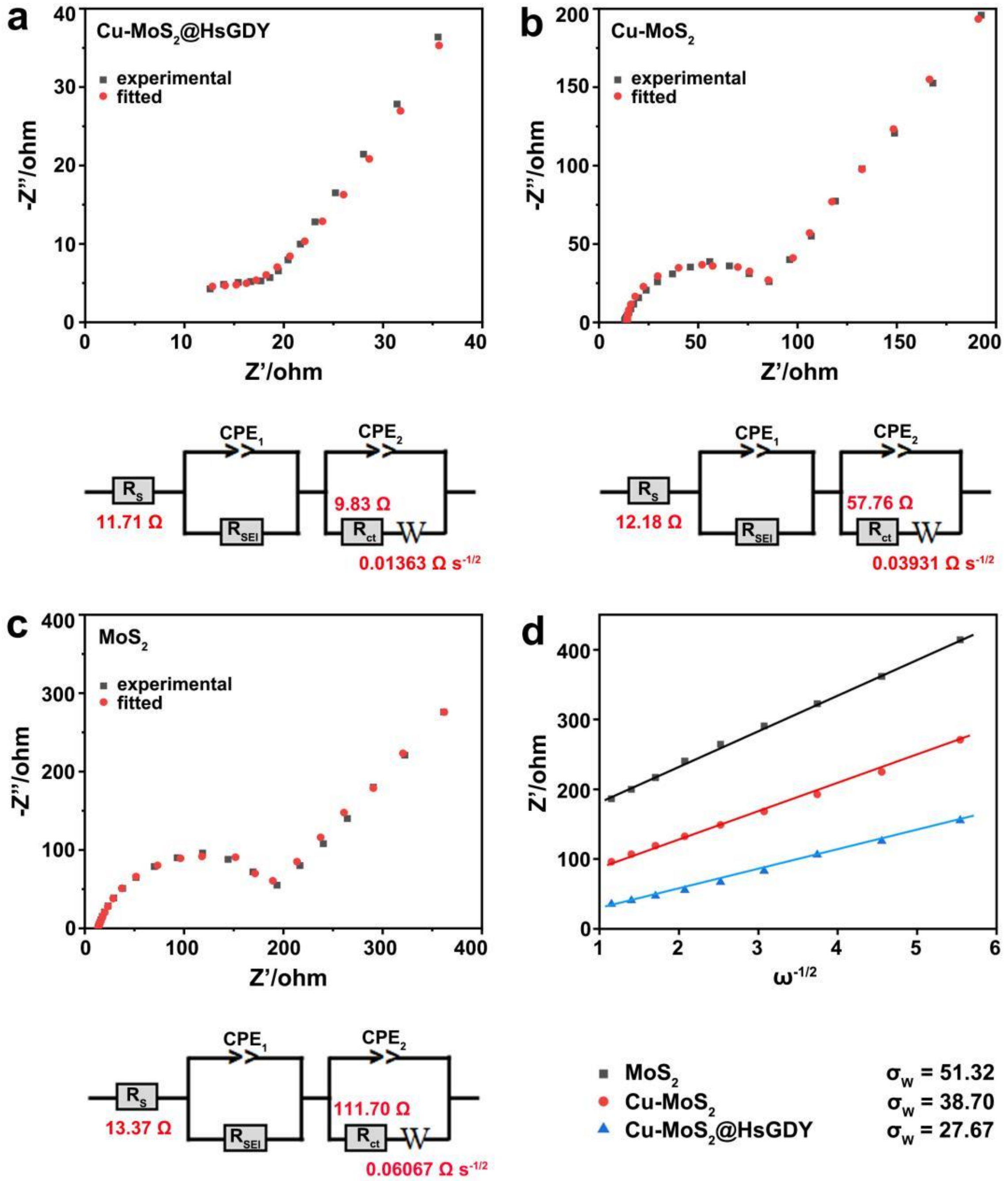

Figure S27. EIS Nyquist plots of Cu-MoS_2@HsGDY (a), Cu-MoS_2 (b), MoS_2 (c), and their corresponding equivalent electrical circuits. d) The Z'-$\omega^{-1/2}$ fitted plots of Cu-MoS_2@HsGDY, Cu-MoS_2 and MoS_2 in the Warburg region, where the corresponding slops are the σ_W values.

As shown in the Nyquist profiles, when applied as cathode materials for magnesium battery, all of the MoS_2, Cu-MoS_2 and Cu-MoS_2@HsGDY demonstrate a semicircle in the high-frequency region and an oblique line in the low-frequency zone, respectively. It is believed that the diameter of the semicircle represents the charge transfer resistance (R_{ct}), while the slope of the oblique line is associated with the Warburg impendence (Z_w), which is further related to the diffusion of the Mg^{2+} ions in the electrode materials. Apparently, a Nyquist plot with a smaller semicircle in high-frequency region and a higher slopes in Warburg region is achieved in order of MoS_2, Cu-MoS_2 and Cu-MoS_2@HsGDY, which suggest both improved charge transfer resistance and Mg^{2+} diffusion in the electrode materials contributed from Cu heteroatoms and HsGDY coating layers. To make a clear understanding, the Mg^{2+} diffusion coefficient (D_{Mg2+}) is utilized to quantify their comparative kinetic effectiveness. In details, the D_{Mg2+} value could be calculated based on the following equations[5,6]:

$$D_{Mg^{2+}} = \frac{R^2T^2}{2n^4F^4\sigma_W^2A^2C^2} \quad (1)$$

$$Z' = R + \sigma_W\omega^{-1/2} \quad (2)$$

where R, T, n, F, σ_W, A and C are the gas constant, absolute kelvin temperature, charge transfer number in the redox reaction, Faraday constant, Warburg coefficient, electrode's surface area and Mg^{2+} concentration in the electrode material, respectively. Given that R, T, n, F, A and C are constant for all of MoS_2, Cu-MoS_2 and Cu-MoS_2@HsGDY, the D_{Mg2+} value is inversely proportional to the square of Warburg coefficient (σ_W) according to **equation 1**. To evaluate the σ_W values, the Z'-$\omega^{-1/2}$ plots are fitted according to **equation 2** at the low frequency zone of the corresponding Nyquist plots. As shown in Figure S27d, the σ_W values of MoS_2, Cu-MoS_2 and

Cu-MoS_2@HsGDY are 51.32, 38.70 and 27.67, respectively. As a result, after doping with Cu heteroatoms, the Mg^{2+} diffusion coefficient for Cu-MoS_2 is 1.76 times higher than that of MoS_2. When Cu-MoS_2 is further encapsulated in the HsGDY coating, the Mg^{2+} diffusion coefficient is nearly doubled in the mode of Cu-MoS_2@HsGDY than that of Cu-MoS_2.

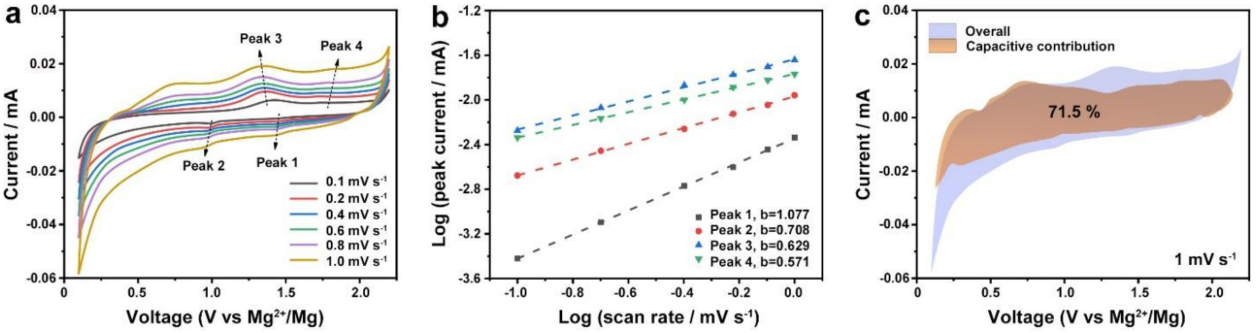

Figure S28. a) CV curves of Cu-MoS_2@HsGDY nanocapsules at different scan rates from 0.1 to 1.0 mV s^{-1}. b) The calculated b-values through the relationship between the peak currents and scan rates. c) Normalized capacitive contribution of Cu-MoS_2@HsGDY nanocapsules at 1 mV s^{-1}. As shown in Figure S28a, the currents of the main redox peaks steadily increase with the growth of the scan rates from 0.1 to 1.0 mV s^{-1}, which suggest the fast kinetics during the Mg^{2+} intercalation/deintercalation process. Since the relationship between the scan rate (v) and current (i) follow the power law as bellow:

$$i = av^b \tag{1}$$

$$logi = blogv + loga \tag{2}$$

It is proposed that the corresponding b value can draw a distinction between the diffusion-controlled process (b≈0.5) and the surface-capacitive behaviour (b≈1.0). As a result, the calculated b values of the main redox peaks are in between 0.5-1.0 (Figure S28b), which suggest

the combined contribution from both the diffusion-controlled process and the surface-capacitive behaviour of the hierarchical Cu-MoS_2@HsGDY nanocapsule. Accordingly, the capacitive contribution covers 71.5% of the CV region at the scan rate of 1.0 mV s^{-1} (Figure S28c). The dominated capacitive contribution suggest that the Faraday reaction takes place on the surface of the Cu-MoS_2@HsGDY, which is easily accessed by Mg^{2+} ions in the electrolyte. This electrochemical behaviour is consistent with the fluffy structure of Cu-MoS_2 with both enlarged interlayer-distance and dominated active Cu-Mo-S phase in basal planes, which provide abundant accessible sites and lower energy barrier for Mg^{2+} ions storage.

Table S1 Comparison of the present work with other MoS_2-based cathodes for Mg^{2+} ion storage.

**Materials**	**electrolyte**	**Voltage (V vs Mg/Mg^{2+})**	**Current density (mA g^{-1})**	**Capacity (mAh·g^{-1}/cycle)**	**Ref.**
Cu-MoS_2@HsGDY Nanocapsule	0.25 M $MgCl_2$: $AlCl_3$ (1:2) in DME	0.1-2.2	50<br>200<br>500	148.5/200th<br>100/300th<br>85.5/300th	Our work
MoS_2@C-PNR	0.4 M Magnesium tetrakis(hexafluoroisopropyloxy)borate in DME	0.01-2.5	50<br>200<br>500	90/100st<br>48/200th<br>38/200th	7
PVP–MoS_2 nanospheres	0.25M APC	0.2-2.0	20	132/100th	8

Poly(ethylene oxide)-MoS_2	$[Mg_2Cl_3]^+[AlCl_2Ph_2]^-$/THF	0.2-2.0	5	75	9
Graphene-like MoS_2/graphene	0.4 M $(PhMgCl)_2$-$AlCl_3$/THF	0-2.2	20	82.5/50th	10
Graphene-like MoS_2	$Mg(AlCl_3Bu)_2$/THF	0-3.0	20	161/50th	11
Cu_2MoS_2 Nanocages	G4-HMDS	0.1-2.7	50<br>200<br>500	180/50th<br>90/200th<br>25/2500th	12

Reference

1. Jiang, L.; Zhu, Y. J. Cu_2S Nanostructures Prepared by Cu-Cysteine Precursor Templated Route. *Mater. Lett.* **2009**, *63*, 1935.
2. He, J.; Wang, N.; Cui, Z.; Du, H.; Fu, L.; Huang, C.; Yang, Z.; Shen, X.; Yi, Y.; Tu, Z.; Li, Y. Hydrogen Substituted Graphdiyne as Carbon-Rich Flexible Electrode for Lithium and Sodium Ion Batteries. *Nat. Commun.* **2017**, *8*, 1172.
3. Gao, X.; Zhu, Y.; Yi, D.; Zhou, J.; Zhang, S.; Yin, C.; Ding, F.; Zhang, S.; Yi, X.; Wang, J.; Tong, L.; Han, Y.; Liu, Z.; Zhang, J. Ultrathin Graphdiyne Film on Graphene Through Solution-Phase Van Der Waals Epitaxy. S*ci. Adv.* **2018**, *4*, eaat6378
4. Chemical Kinetics and Reaction Mechanisms, McGraw, New York, NY, **2002**, 1–14.

5. Yao, K.; Xu, Z.; Huang, J.; Ma, M.; Fu, L.; Shen, X.; Li, J.; Fu, M. Bundled Defect-Rich MoS_2 for a High-Rate and Long-Life Sodium-Ion Battery: Achieving 3D Diffusion of Sodium Ion by Vacancies to Improve Kinetics. *Small*, **2019**, *15*, 1805405.
6. Ye, W.; Wu, F.; Shi, N.; Zhou, H.; Chi, Q.; Chen, W.; Du, S.; Gao, P.; Li, H.; Xiong S. Metal-Semiconductor Phase Twinned Hierarchical MoS_2 Nanowires with Expanded Interlayers for Sodium-Ion Batteries with Ultralong Cycle Life. *Small* **2020**, *16*, 1906607.
7. Li, Z.; Mu, X.; Zhao, Z.; Diemant, T.; Behm, R. J.; Kübel, C.; Fichtner, M. Fast Kinetics of Multivalent Intercalation Chemistry Enabled by Solvated Magnesium-Ions into Self-Established Metallic Layered Materials. *Nat. Commun*. **2018**, *9*, 5115.
8. Wu, C.; Zhao, G.; Gong, S.; Zhang, N.; Sun, K. PVP Incorporated MoS_2 as A Mg Ion Host with Enhanced Capacity and Durability. *J. Mater. Chem. A*, **2019**, *7*, 4426.
9. Liang, Y.; Yoo, H. D.; Li, Y.; Shuai, J.; Calderon, H. A.; Hernandez, F. C. R.; Grabow, L. C.; Yao, Y. Interlayer-Expanded Molybdenum Disulfide Nanocomposites For Electrochemical Magnesium Storage. *Nano Lett*. **2015**, *15*, 2194.
10. Liu, Y.; Fan, L.-Z.; Jiao, L. Graphene Intercalated in Graphene-Like MoS_2: A Promising Cathode for Rechargeable Mg Batteries. *J. Power Sources*, ***2017***, *340*, 104.
11. Liang, Y.; Feng, R.; Yang, S.; Ma, H.; Liang, J.; Chen, J. Rechargeable Mg Batteries with Graphene-Like MoS_2 Cathode and Ultrasmall Mg Nanoparticle Anode. *Adv. Mater*. **2011**, *23*, 640.
12. Zhang, Y.; Li, T.; Cao, S.; Luo, W.; Xu, F. Cu_2MoS_4 Hollow Nanocages with Fast and Stable Mg^{2+}-Storage Performance. *Chem. Eng. J.* **2020**, *387*, 124125.
